# A Comprehensive Analysis for Expression, Diagnosis, and Prognosis of m^5^C Regulator in Breast Cancer and Its ncRNA–mRNA Regulatory Mechanism

**DOI:** 10.3389/fgene.2022.822721

**Published:** 2022-06-22

**Authors:** Jingxing Liu, Shuyuan Xiao, Jing Chen, Weiyang Lou, Xu Chen

**Affiliations:** ^1^ Department of Intensive Care Unit, Changxing People’s Hospital of Zhejiang, Huzhou, China; ^2^ Department of Anesthesiology, The First Affiliated Hospital, College of Medicine, Zhejiang University, Hangzhou, China; ^3^ Department of Oncology, The First Affiliated Hospital of Jiaxing University, Jiaxing, China; ^4^ Department of Breast Surgery, The First Affiliated Hospital, College of Medicine, Zhejiang University, Hangzhou, China; ^5^ Emergency & Intensive Care Unit Center, Department of Intensive Care Unit, Zhejiang Provincial People’s Hospital, Affiliated People’s Hospital, Hangzhou Medical College, Hangzhou, China

**Keywords:** breast cancer, microRNA (miRNA), 5-methylcytosine (m^5^C), DNA methyltransferase 3 beta, ALYREF, bioinformatic analysis

## Abstract

Recent studies have well demonstrated that 5-methylcytosine (m^5^C) regulators play pivotal roles in pathological conditions, including cancer. This study first tried to identify potential 5-methylcytosine (m^5^C) regulators in breast cancer by combination of expression, diagnosis, and survival analyses, and then established an ncRNA–mRNA network accounting for m^5^C regulators’ roles in breast cancer. Among 13 m^5^C regulators, DNMT3B and ALYREF were significantly upregulated in breast cancer and their high expression indicated unfavorable prognosis. Both DNMT3B and ALYREF possessed the statistical abilities to distinguish breast cancer from normal breast samples. Moreover, five potential upstream miRNAs (let-7b-5p, miR-195-5p, miR-29a-3p, miR-26a-5p, and miR-26b-5p) of m^5^C regulators could not only serve as independent prognostic predictors but also together made up a promising miRNA prognostic signature in breast cancer. Next, upstream potential lncRNAs of the five miRNAs were predicted and analyzed. Pathway enrichment analysis revealed that the target genes of these miRNAs were markedly enriched in some cancer-related pathways, and further investigation indicated *VEGFA* and *EZH2* were found to be the most potential target genes in the m^5^C regulators-related ncRNA–mRNA network in breast cancer. These findings comprehensively provided key clues for developing m^5^C regulators-related effective therapeutic targets and promising diagnostic biomarkers in breast cancer.

## Introduction

It has been widely acknowledged that epigenetic dysregulation partially leads to the occurrence and progression of a variety of human disorders, including malignancies (Koschmieder and Vetrie, 2018; Nakamura et al., 2019). Traditional epigenetic modifications contain DNA methylation, histone modification, and chromatin remodeling (Dawson and Kouzarides, 2012). Recently, the focus on epigenetic research has shifted from DNA to RNA (Wang P et al., 2020). To date, more than 250 types of RNA modification have been identified, among which N6-methyladenosine (m6A) is the most prevalent RNA modification of internal mRNA, and its dysregulation has been found to be closely linked to carcinogenesis (Li et al., 2019). In addition to m6A, 5-methylcytosine (m^5^C) is another RNA modification, which is commonly appeared in mRNAs, tRNAs, and rRNAs (Helm, 2006; Schaefer et al., 2009).

Similar to DNA or protein modification, RNA methylation is also modulated by various types of regulators, such as methyltransferases (“writers”), RNA binding proteins (“readers”), and demethylases (“erasers”) (Li et al., 2019; Boo and Kim, 2020). For m^5^C, 11 “writers” (consisting of NSUN1-7, DNMT1-2, DNMT3A, and DNMT3B), 1 “eraser” (TET2), and 1 “reader” (ALYREF) have been identified (He et al., 2020). Some of these m^5^C regulators have been found to be correlated with cancer initiation and development. For instance, Sun et al. (2020) indicated that NSUN2-mediated m^5^C modification of H19 was correlated with poor differentiation of hepatocellular carcinoma; Jiang et al. (2020) suggested that NSUN5 was overexpressed in colorectal cancer and it promoted proliferation and cell cycle progression of colorectal cancer. However, a comprehensive study focusing on m^5^C regulators in breast cancer remains ambiguous. Furthermore, the miRNA–mRNA network related to m^5^C regulators in breast cancer is also absent.

In this study, we first overviewed the expression profiles, prognostic and diagnostic values of m^5^C regulators in breast cancer. Then, we successively predicted and analyzed the upstream potential binding miRNAs of m^5^C regulators in breast cancer. Next, a miRNA prognostic signature in breast cancer was established. The upstream lncRNAs that could potentially bind to miRNAs were also predicted. Subsequently, downstream target genes of potential miRNAs were forecasted and analyzed. Consequently, an m^5^C regulators-related miRNA–mRNA regulatory network was constructed in breast cancer. These current findings may provide key roles in seeking and developing promising biomarkers and therapeutic targets for breast cancer patients.

## Results

### Overview Expression, Prognosis, and Diagnosis of m^5^C Regulators in Breast Cancer

**FIGURE 1:**
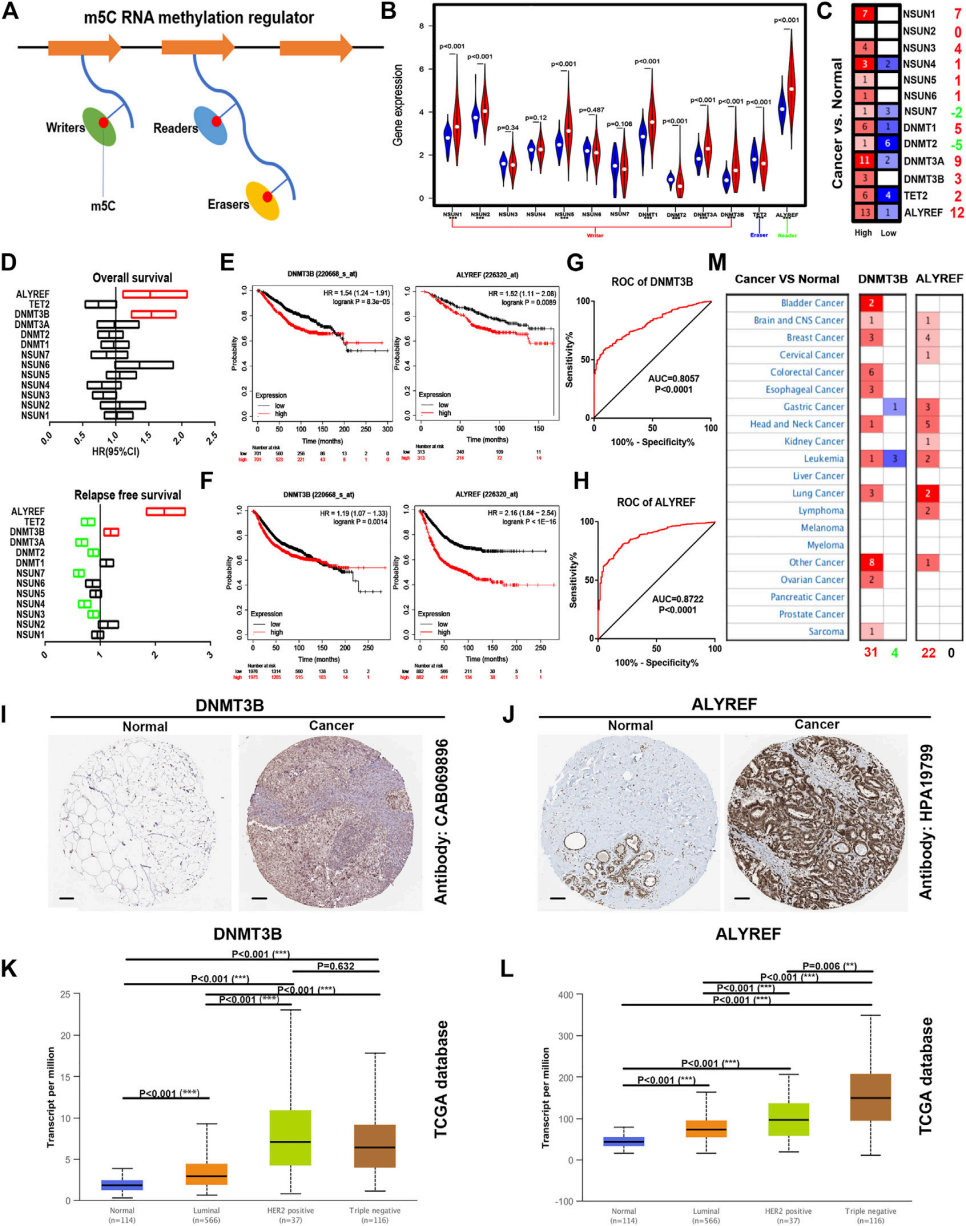
Overview of expression, diagnostic, and prognostic values of m^5^C regulators in breast cancer. **(A)** Diagram of m^5^C regulators. **(B)** Expression of m^5^C regulators in TCGA breast cancer cohort. Blue: normal samples; red: cancer samples. **(C)** Expression of m^5^C regulators determined by the Oncomine database. **(D)** Survival analysis (including overall survival and relapse-free survival) for m^5^C regulators in breast cancer using the Kaplan–Meier plotter. **(E)** Prognostic values of DNMT3B in breast cancer. **(F)** Prognostic values of ALYREF in breast cancer. **(G)** ROC curve of DNMT3B in breast cancer. **(H)** ROC curve of ALYREF in breast cancer. The protein level of DNMT3B **(I)** or ALYREF **(J)** in breast cancer detected by Human Protein Atlas database. Scale bar: 100 um. **(K)** Expression of DNMT3B in breast cancer among various stages. **(L)** Expression of ALYREF in breast cancer among various stages. **(M)** **(I)** Expression of DNMT3B and ALYREF across different cancer types by the Oncomine database. **p < 0.01; ***p < 0.001.

It has been well known that m^5^C regulators can be generally divided into three classes, namely, “writer” (NSUN1-7, DNMT1-2, DNMT3A, and DNMT3B), “eraser” (TET2), and “reader” (ALYREF) as vividly presented in Figure 1A. To explore their underlying roles of these m^5^C regulators in breast cancer, we first determined their expression levels using TCGA breast cancer data. As shown in Figure 1B, expression of NSUN1, NSUN2, NSUN5, DNMT1, DNMT3A, DNMT3B, and ALYREF was significantly increased, but DNMT2 and TET2 expression was markedly decreased in breast cancer tissues when compared with normal breast tissues. For NSUN3, NSUN4, NSUN6, and NSUN7, no statistical differences were observed between breast cancer samples and control samples. Moreover, we found that, among these m^5^C regulators, ALYREF was most highly expressed in breast cancer. Next, Oncomine analysis was employed to further assess expression of m^5^C regulators in breast cancer. The result demonstrated that most of these m^5^C regulators, except NSUN2, NSUN7, and DNMT2, were overexpressed in breast cancer (Figure 1C). Subsequently, the prognostic values of m^5^C regulators in breast cancer were evaluated, containing two indices overall survival (OS) and relapse-free survival (RFS), as presented in Figure 1D. Intriguingly, only breast cancer patients with higher expression of DNMT3B and ALYREF had poorer OS and RFS (Figures 1E,F). By combination of expression and survival analyses, DNMT3B and ALYREF were selected for subsequent investigation. ROC curve analysis was conducted to determine the diagnostic values of DNMT3B and ALYREF in breast cancer. As shown in Figures 1G,H, both DNMT3B and ALYREF possessed the significant abilities to distinguish breast cancer tissues from normal breast tissues. Moreover, DNMT3B and ALYREF protein levels in breast cancer tissues were also obviously higher than that in normal breast tissues (Figures 1I,J). Based on molecular characteristics, breast cancer can be classified into three different subtypes, consisting of luminal, HER2 positive, and triple-negative breast cancer. Thus, we also assessed the expression of DNMT3B and ALYREF in luminal, HER2 positive, and triple negative breast cancer. As presented in Figure 1K, DNMT3B expression in HER2 positive and triple-negative breast cancer were higher than that in luminal breast cancer. For ALYREF, its expression in triple-negative breast cancer was highest and in luminal breast cancer was lowest. All these findings together indicate that DNMT3B and ALYREF may be two most potential oncogenes in breast cancer among all these m^5^C regulators.

### Identification of Upstream miRNAs of m^5^C Regulators in Breast Cancer

**FIGURE 2:**
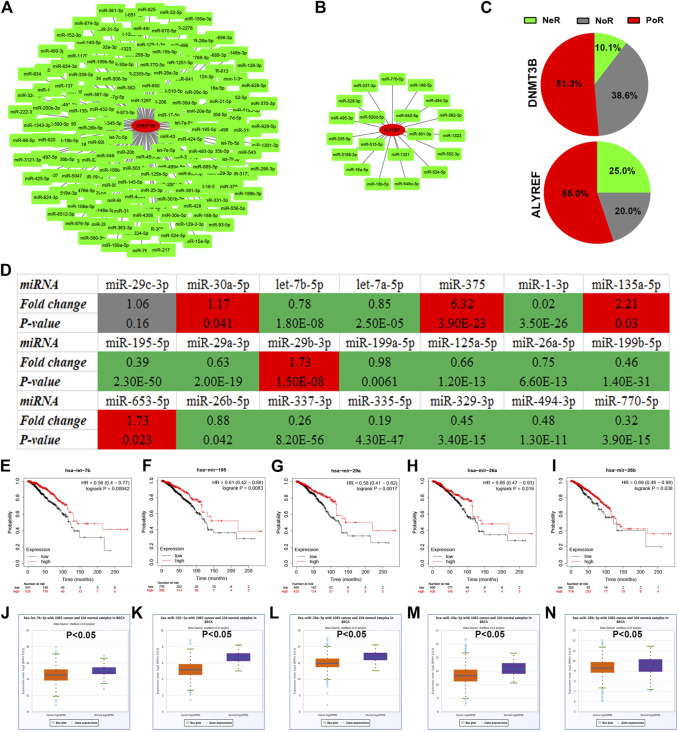
Identification of potential miRNAs of m^5^C regulators in breast cancer. **(A)** miRNA–DNMT3B network. **(B)** miRNA-ALYREF network. **(C)** Distribution of expression correlation among miRNA-DNMT3B or miRNA-ALYREF pairs. NeR: negative correlation; PoR: positive correlation; NoR: no significant correlation. **(D)** Expression landscape of candidate miRNAs of DNMT3B or ALYREF in breast cancer. The prognostic values of let-7b-5p **(E)**, miR-195-5p **(F)**, miR-29a-3p **(G)**, miR-26a-5p **(H)**, and miR-26b-5p **(I)** in breast cancer. The expression levels of let-7b-5p **(J)**, miR-195-5p **(K)**, miR-29a-3p **(L)**, miR-26a-5p **(M)**, and miR-26b-5p **(N)** in breast cancer.

Next, we predicted upstream miRNAs that could bind to DNMT3B or ALYREF using the starBase database. A total of 158 and 20 miRNAs were found to be potentially binding to DNMT3B or ALYREF, respectively (Supplementary Table S1). For better visualization, DNMT3B-miRNA and ALYREF-miRNA networks were established as shown in Figures 2A,B. Based on the action mechanism of miRNA, there should be negative relationship between DNMT3B/ALYREF and their corresponding miRNAs. Thus, expression correlation of DNMT3B/ALYREF with miRNAs in breast cancer was determined (Supplementary Table S2). Further analysis revealed that more than half of DNMT3B/ALYREF-miRNA pairs showed positive expression correlation (51.3% for DNMT3B and 55.0% for ALYREF) in breast cancer, but only 10.1 and 25.0% pairs presented negative expression relationship for DNMT3B and ALYREF, respectively (Figure 2C). Subsequently, miRNAs in these DNMT3B/ALYREF-miRNA pairs with negative expression correlation were chosen for expression analysis in breast cancer. The expression landscape of these miRNAs in breast cancer is vividly shown in Figure 2D. The result suggested that 15 of 21 miRNAs were significantly downregulated in breast cancer when compared with normal controls. Finally, survival analysis was conducted to evaluate the prognostic values of the 15 miRNAs in breast cancer. As shown in Figures 2E–I, among these miRNAs, only high expression of five miRNAs (consisting of let-7b-5p, miR-195-5p, miR-29a-3p, miR-26a-5p, and miR-26b-5p) indicated favorable prognosis in breast cancer. Moreover, expression levels of the five miRNAs in breast cancer are also presented in Figures 2J–N. Taken correlation analysis, expression analysis, and survival analysis into consideration, let-7b-5p, miR-195-5p, miR-29a-3p, miR-26a-5p, and miR-26b-5p may be five most potential upstream tumor suppressive miRNAs of m^5^C regulators in breast cancer. By matching with DNMT3B/ALYREF-miRNA pairs, we found that all the five miRNAs could only potentially bind to DNMT3B.

### Prediction of Upstream lncRNAs of miRNAs in Breast Cancer

To further find the upstream possible lncRNAs of the five miRNAs (let-7b-5p, miR-195-5p, miR-29a-3p, miR-26a-5p, and miR-26b-5p), two online databases, consisting of starBase and miRNet, were employed. By intersection of the analytic results from starBase and miRNet databases, 53, 112, 52, 43, and 43 lncRNAs were, respectively, forecasted to potentially bind to let-7b-5p, miR-195-5p, miR-29a-3p, miR-26a-5p, and miR-26b-5p as listed in Supplementary Table S3.

### Construction of a Potential miRNA Prognostic Signature in Breast Cancer

**FIGURE 3:**
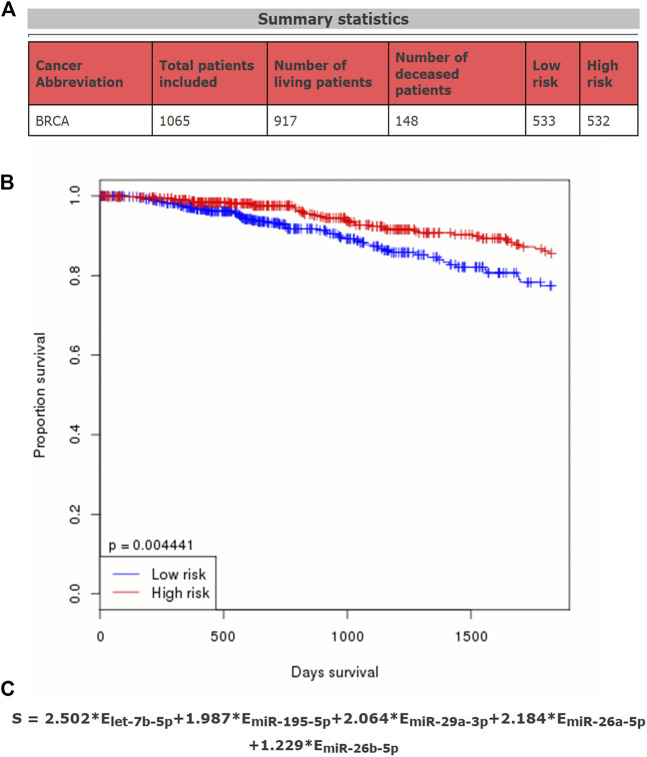
Establishment of a potential prognostic signature based on five miRNAs (let-7b-5p, miR-195-5p, miR-29a-3p, miR-26a-5p, and miR-26b-5p) in breast cancer. **(A)** Summary statistics of data used in this prediction model. **(B)** Survival curve of the constructed miRNA signature in breast cancer. According to the median expression of miRNAs calculated by the formula, the total breast cancer patients can be divided into two groups, including high-risk group and low-risk group. **(C)** Calculating formula of this miRNA prognostic signature.

Our data suggested that each of the five potential miRNAs (let-7b-5p, miR-195-5p, miR-29a-3p, miR-26a-5p, and miR-26b-5p) could be used to independently predict prognosis of breast cancer patients. We then further determined whether an miRNA-related prognostic model, consisting of the five potential miRNAs, could be constructed in breast cancer. A total of 1065 TCGA breast cancer patients, containing 917 living patients and 148 deceased patients, were employed (Figure 3A). As shown in Figure 3B, high expression of five miRNAs’ sum indicated good prognosis in breast cancer (*p*-value = 0.004441). The expression sum of this model was calculated by the following formula: 2.502*E_let-7b-5p_ + 1.987*E_miR-195-5p_ + 2.064*E_miR-29a-3p_ + 2.184*E_miR-26a-5p_ + 1.229*E_miR-26b-5p_ (Figure 3C). The established miRNA prognostic signature might be utilized as a potential model for predicting prognosis of patients with breast cancer.

### Enrichment Analysis and Protein–Protein Interaction Network Analysis

**FIGURE 4:**
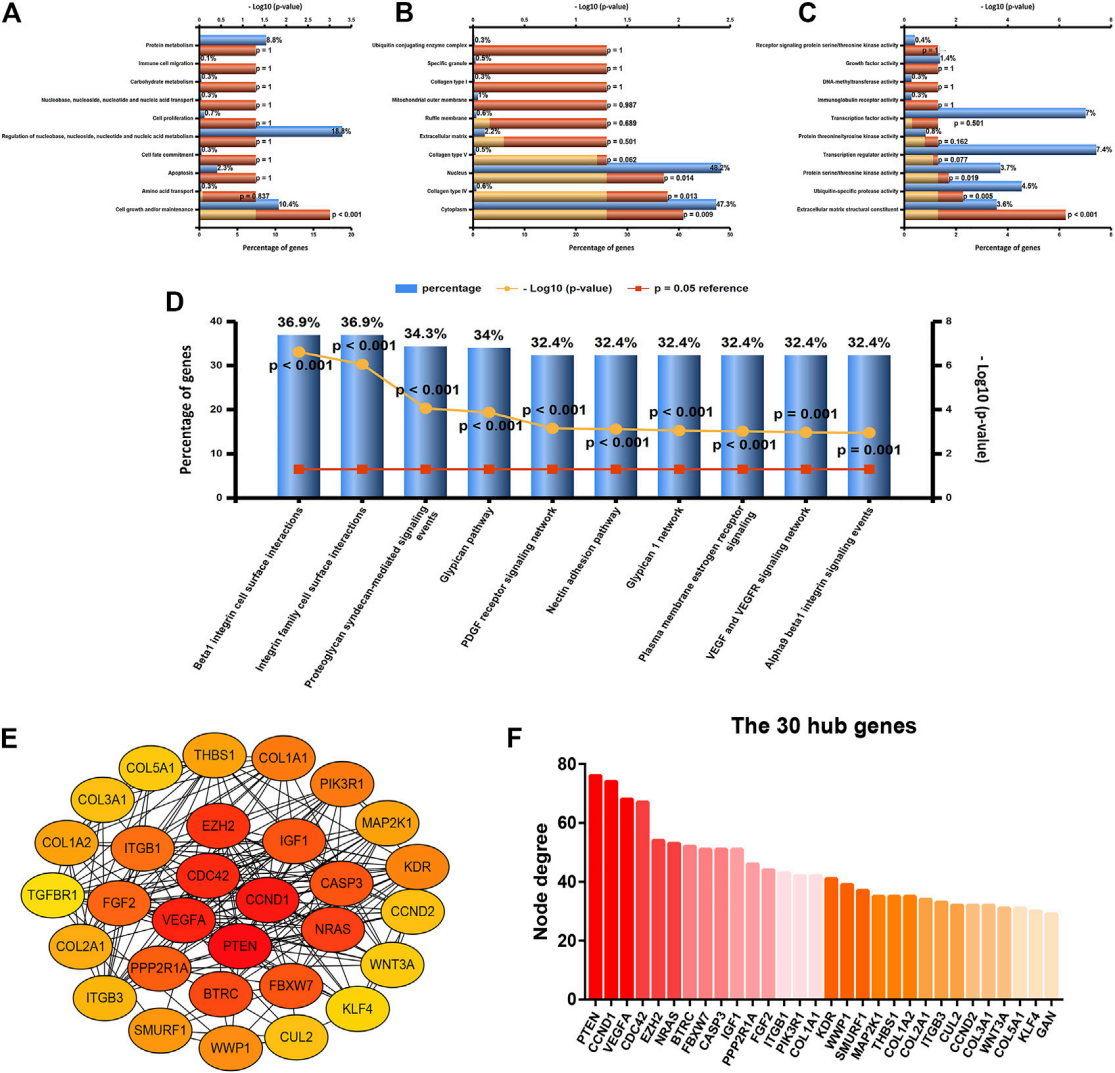
Enrichment analysis and protein–protein interaction (PPI) network analysis for the target genes of five miRNAs (let-7b-5p, miR-195-5p, miR-29a-3p, miR-26a-5p, and miR-26b-5p). **(A)** Top 10 enriched biological process (BP) items analyzed by FunRich. **(B)** Top 10 enriched cellular component (CC) items analyzed by FunRich. **(C)** Top 10 enriched molecular function (MF) items analyzed by FunRich. **(D)** Top 10 enriched biological pathway items analyzed by FunRich. **(E)** PPI sub-network of top 30 hub genes according to node degree. **(F)** Top 30 hub genes ranked by node degree.

To further explore the underlying functions, downstream target genes of let-7b-5p, miR-195-5p, miR-29a-3p, miR-26a-5p, and miR-26b-5p were first predicted. A total of 758 target genes, involving 164 of let-7b-5p, 288 of miR-195-5p, 237 of miR-195-5p, 121 of miR-26a-5p, and 104 of miR-26b-5p, were forecast as listed in Supplementary Table S4. Gene Ontology function annotation revealed that these target genes were significantly enriched in cell growth and/or maintenance for biological process category (Figure 4A), cytoplasm, collagen type IV, and nucleus for cellular component category (Figure 4B), and extracellular matrix structural constituent, ubiquitin-specific protease activity, and protein serine/threonine kinase activity for molecular function category (Figure 4C). Next, pathway enrichment analysis for these target genes demonstrated that they were obviously enriched in a lot of cancer-related pathways, such as beta-1 integrin surface interactions, integrin family cell surface interactions, and VEGF and VEGFR signaling networks (Figure 4D). In order to have a good command of the interactions among these target genes, PPI network analysis was carried out using STRING, after which the top 30 hub genes were identified based on node degree, and a sub-PPI network was established by usage of Cytoscape software (Figure 4E). As shown in Figure 4F, among this PPI network, PTEN, CCND1, VEGFA, CDC42, and EZH2 were ranked as the top five hub genes, which may function as key genes in the m^5^C regulators-related miRNA–mRNA regulatory network in breast cancer.

### Establishment of a DNMT3B-Related miRNA–mRNA Network in Breast Cancer

**FIGURE 5:**
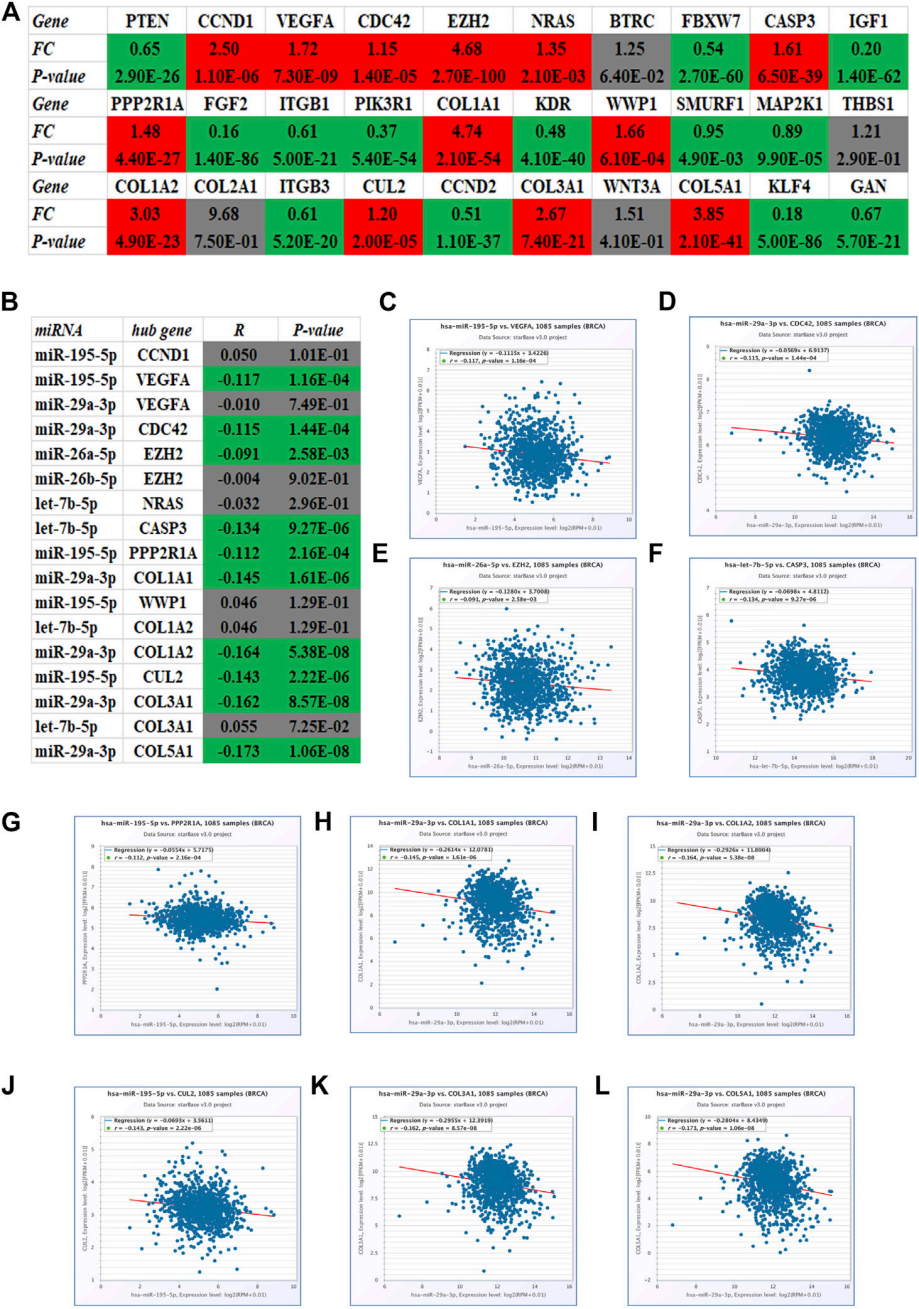
Identification of potential target genes of five miRNAs (let-7b-5p, miR-195-5p, miR-29a-3p, miR-26a-5p, and miR-26b-5p) in breast cancer using starBase. **(A)** Expression landscape of top 30 hub genes in breast cancer. **(B)** Correlation analysis for the candidate miRNA-hub gene pairs in breast cancer. The expression relationship of miR-195-5p/VEGFA **(C)**, miR-29a-3p/CDC42 **(D)**, miR-26a-5p/EZH2 **(E)**, let-7b-5p/CASP3 **(F)**, miR-195-5p/PPP2R1A **(G)**, miR-29a-3p/COL1A1 **(H)**, miR-29a-3p/COL1A2 **(I)**, miR-195-5p/CUL2 **(J)**, miR-29a-3p/COL3A1 **(K)**, and miR-29a-3p/COL5A1 **(L)** pairs in breast cancer.

**FIGURE 6:**
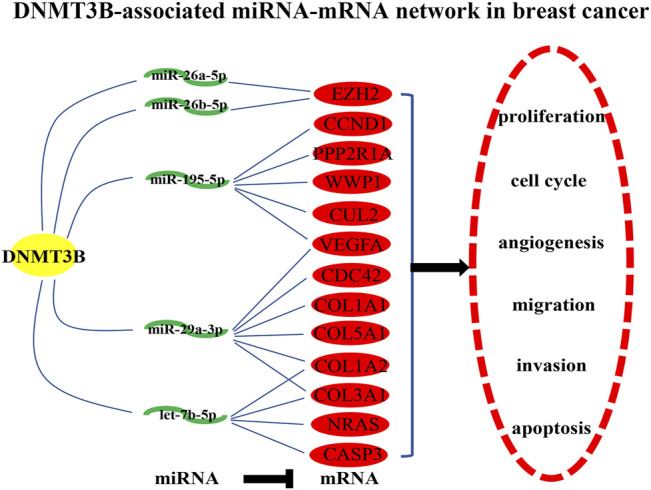
Construction of a potential DNMT3B-associated miRNA–mRNA network in breast cancer.

Identically, there should be negative relationship between let-7b-5p, miR-195-5p, miR-29a-3p, miR-26a-5p, and miR-26b-5p and their respective target genes in breast cancer. Therefore, expression levels of the top 30 hub genes in breast cancer were detected using TCGA breast cancer data. As presented in Figure 5A, only 13 of 30 hub genes (CCND1, VEGFA, CDC42, EZH2, NRAS, CASP3, PPP2R1A, COL1A1, WWP1, COL1A2, CUL2, COL3A1, and COL5A1) were significantly upregulated in breast cancer, indicating that they might be the potential target genes of let-7b-5p, miR-195-5p, miR-29a-3p, miR-26a-5p, and miR-26b-5p. Moreover, the expression correlation of these miRNA-target gene pairs (N = 17) in breast cancer were evaluated. As shown in Figures 5C–L, 10 of 17 miRNA-target gene pairs had negative expression relationship, including miR-195-5p/VEGFA, miR-29a-3p/CDC42, miR-26a-5p/EZH2, let-7b-5p/CASP3, miR-195-5p/PPP2R1A, miR-29a-3p/COL1A1, miR-29a-3p/COL1A2, miR-195-5p/CUL2, miR-29a-3p/COL3A1, and miR-29a-3p/COL5A1 pairs. Finally, a potential DNMT3B-related miRNA–mRNA network in breast cancer was constructed (Figure 6).

### Screening of VEGFA and EZH2 as Two Most Potential Targets in the DNMT3B-Related miRNA–mRNA Network

**FIGURE 7:**
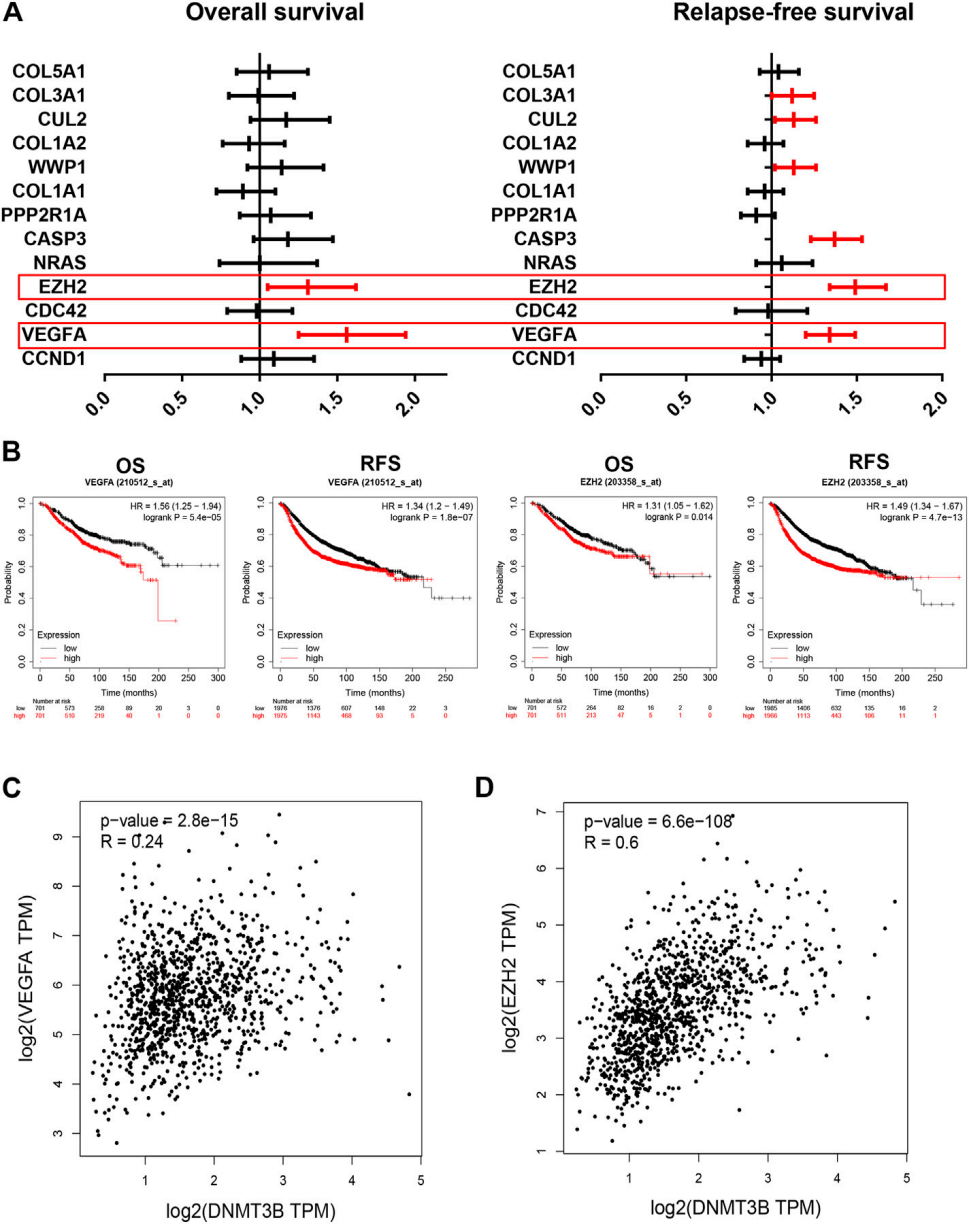
Identification of VEGFA and EZH2 as the most potential downstream target genes in the established DNMT3B-associated miRNA–mRNA network. Overall survival **(A)** and relapse-free survival **(B)** analyses for 10 genes of interest in breast cancer. **(B)** Prognostic values of VEGFA and EZH2 in breast cancer determined by the Kaplan–Meier plotter. DNMT3B expression was significantly positively correlated with the expression of VEGFA **(C)** or EZH2 **(D)** in breast cancer assessed by GEPIA.

Furthermore, survival analysis for the 10 target genes involving in the established DNMT3B-related miRNA–mRNA network was performed using the Kaplan–Meier plotter. As shown in Figure 7A, only high expression of two genes, including VEGFA and EZH2, indicated unfavorable overall survival of breast cancer patients. For relapse-free survival, breast cancer patients with higher expression of VEGFA, EZH2, CASP3, WWP1, CUL2, and COL3A1 had poorer prognosis (Figure 7A). By combination of overall survival and relapse-free survival, we found that only VEGFA and EZH2 were commonly appeared in significant OS gene set and RFS gene set. The corresponding survival plots of VEGFA and EZH2 are presented in Figure 7B. Moreover, expression correlation analysis suggested that DNMT3B was markedly positively associated with VEGFA and EZH2 in breast cancer (Figures 7C,D). Conclusively, VEGFA and EZH2 may be the most potential targets in the DNMT3B-related miRNA–mRNA network in breast cancer.

## Discussion

**FIGURE 8:**
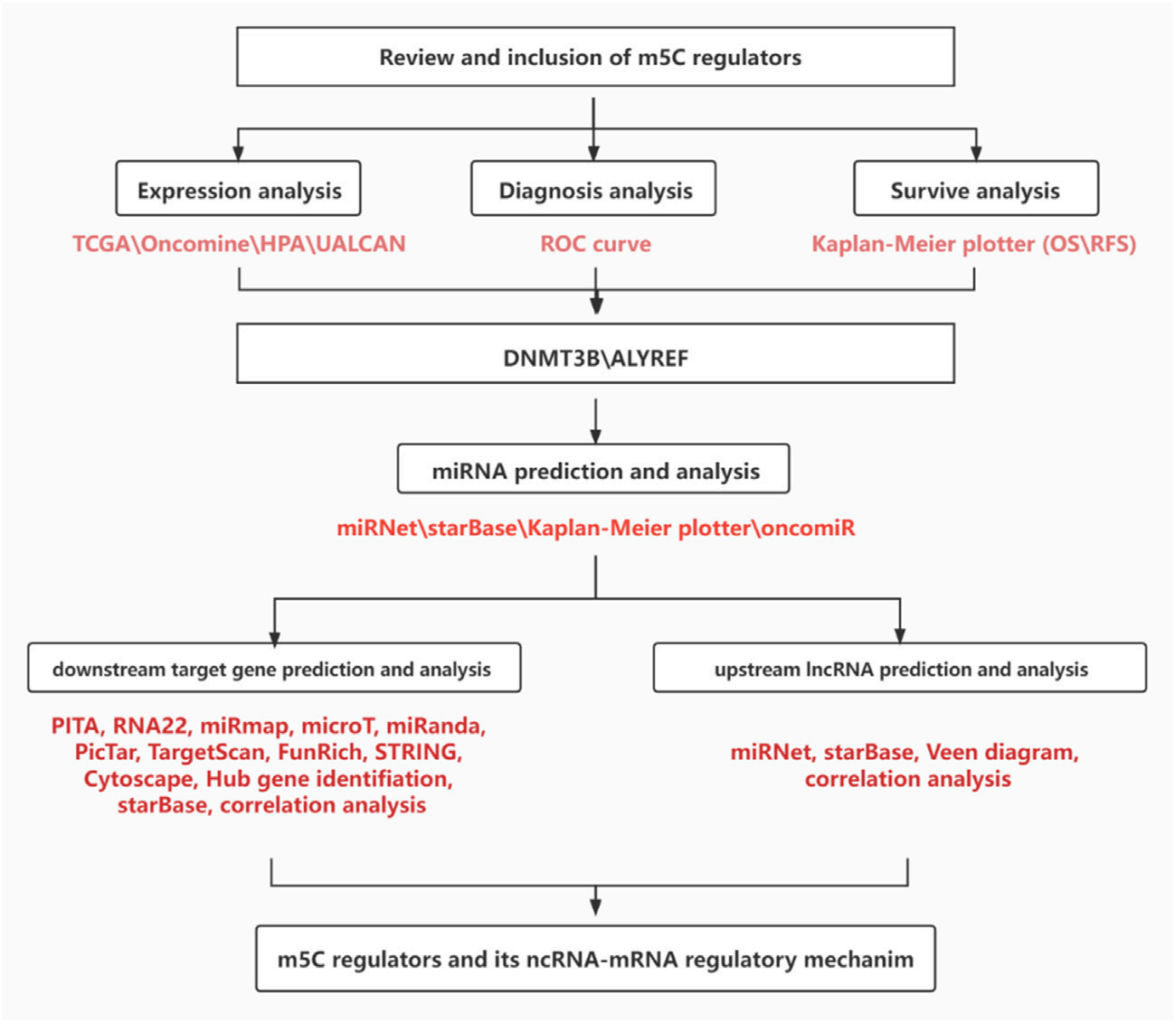
Flow chart of this study.

During the past decade, with advancement of RNA direct sequencing technique, the emerging roles of RNA m^5^C modification in tumorigenesis have been reported (Xue et al., 2020). As mentioned earlier, to date, a total of 13 m^5^C regulators were found. In general, this research aimed to identify one or more potential members among these m^5^C regulators in modulating initiation and progression of breast cancer by performing a series of bioinformatic analyses (Figure 8).

First of all, the expression landscape and prognostic values of m^5^C regulators in breast cancer were overviewed by using multiple databases. DNMT3B and ALYREF might be the most potential functional m^5^C regulators and promising biomarkers in breast cancer. Multiple studies showed that DNMT3B acted as an oncogene in tumorigenesis, including acute myeloid leukemia (Wong et al., 2019), gastric cancer (Li et al., 2016), bladder cancer (Liu et al., 2020), and prostate cancer (Zhu et al., 2020). For ALYREF, researches related to the function and mechanism of ALYREF in human cancers remain absent. Next, ROC curve analysis for DNMT3B and ALYREF revealed their significant diagnostic roles in breast cancer.

It has been widely acknowledged that the miRNA–mRNA regulatory axis has inseparable connection with onset and progression of human malignancies, involving breast cancer (He et al., 2019; Yao et al., 2019; Gao et al., 2020; Lü et al., 2020). Therefore, we further explored the potential miRNA–mRNA regulatory axis contributing to explanation for m^5^C regulators-mediated oncogenic roles in breast cancer. A total of 178 candidate miRNAs binding to DNMT3B or ALYREF were predicted by bioinformatic analysis. Subsequently, by combining expression and survival analyses, five potential miRNAs (including let-7b-5p, miR-195-5p, miR-29a-3p, miR-26a-5p, and miR-26b-5p) have been screened, which were significantly downregulated in breast cancer and their low expression indicated poor prognosis of patients with breast cancer. These miRNAs have been reported to function as tumor suppressive miRNAs in breast cancer. For example, Al-Harbi et al. (2018)showed that let-7b-5p inhibited the cancer-promoting effects of breast cancer-associated fibroblasts through IL-8 suppression ; Marques et al. (2018)suggested that miR-195-5p served as a tumor suppressor in invasive breast cancer ); Zhao et al. (2017) found that miR-29a-3p suppressed MCF-7 cell growth by decreasing expression of tumor necrosis factor receptor 1. miR-26a-5p was also confirmed to inhibit breast cancer cell growth by suppression of RNF6 expression (Huang et al., 2019); miR-26b-5p played a suppressive role in inhibiting proliferation of breast cancer cells by negatively regulating CDK8 (Li et al., 2014). These reports together with our previous analytic results indicate that the five miRNAs might play crucial effects in m^5^C regulators-related functions in breast cancer. Moreover, an miRNA-associated signature composed by the five miRNAs also presented a significant predictive effect for prognosis of breast cancer.

Next, the potential downstream molecular mechanism of the five miRNAs was explored by a series of *in silico* analyses. Pathway enrichment analysis revealed that the targets of the five miRNAs were markedly enriched in multiple cancer-related pathways, such as the glypican pathway (Castillo et al., 2016; Guereño et al., 2020) and VEGF and VEGFR signaling networks (Zhang et al., 2020). After conducting PPI network establishment and analysis, PTEN, CCND1, VEGFA, CDC42, and EZH2 were screened as the top five hub genes. Further analysis revealed that VEGFA and EZH2 were negatively correlated with their respective upstream miRNAs in breast cancer and were significantly overexpressed in breast cancer compared with normal breast controls and indicated poor prognosis of patients with breast cancer. Taken all these results into consideration, VEGFA and EZH2 might be the most potential targets involved in the established m^5^C regulators-associated miRNA–mRNA network in breast cancer.

In total, we constructed a potential m^5^C regulator-associated miRNA–mRNA axis in breast cancer, which probes a comprehensive molecular explanation of breast carcinogenesis and provides important clues for seeking promising therapeutics targets and biomarkers in breast cancer. However, these findings were only obtained from pure bioinformatics research and should be further validated by much more basic experiments and clinical trials in the future.

## Materials and Methods

### Oncomine Analysis

The expression of m^5^C regulators in breast cancer was determined using differential expression analysis provided by Oncomine (https://www.oncomine.org/), which is a cancer microarray database and integrated data-mining platform (Rhodes et al., 2004; Rhodes et al., 2007). Fold change (FC) > 1.5, *p*-value < 0.05, and a gene rank in the top 10% were set as the thresholds for selecting the datasets. In addition, the expression levels of DNMT3B and ALYREF across different cancer types were studied.

### Kaplan–Meier Plotter Analysis

The Kaplan–Meier plotter (http://kmplot.com/analysis), capable of accessing the effect of 54,000 genes on survival in more than 20 cancer types, was employed to assess the prognostic values of genes and miRNAs in breast cancer (Györffy et al., 2010; Lou et al., 2020). Log rank *p*-value < 0.05 was considered as statistically significant.

### Human Protein Atlas Analysis

The HPA database (http://www.proteinatlas.org/), a tool for exploring proteomic biomarker, was utilized to analyze the protein level of DNMT3B and ALYREF in breast cancer and normal breast tissues (Pontén et al., 2011).

### UALCAN Analysis

UALCAN (http://ualcan.path.uab.edu/index.html), a portal for facilitating tumor subgroup gene expression, survival analysis, and correlation analysis, was used to determine the expression of DNMT3B and ALYREF in breast cancer based on different molecular subtypes. The statistical difference was also automatically analyzed by UALCAN. *p*-value < 0.05 was regarded as statistically significant.

### Receiver Operator Characteristic Curve Analysis

As we previously described, the ROC curve was introduced to assess the diagnostic abilities of DNMT3B and ALYREF to distinguish breast cancer samples from normal breast samples using TCGA expression data (Wang W et al., 2020). *p*-value < 0.05 was regarded as statistically significant.

### starBase Analysis

starBase (http://starbase.sysu.edu.cn/), a database for exploring microRNA–mRNA interaction maps from Argonaute CLIP-Seq and Degradome-Seq data, was employed to predict the upstream miRNAs that could potentially bind to DNMT3B or ALYREF. The expression levels of predicted miRNAs and hub genes in breast cancer were also detected by starBase. starBase was also used to analyze the expression correlation of miRNA-target gene pairs. *p*-value < 0.05 was regarded as statistically significant.

### OncomiR Analysis

The OncomiR database (http://www.oncomir.org/cgi-bin/dbSearch.cgi), an online resource for exploring miRNA dysregulation in pan cancer, was utilized to evaluate the predictive value of five miRNAs signatures in breast cancer (Wong et al., 2018). TCGA breast cancer expression and survival data were employed to perform this analysis. After entering the five miRNAs’ names into the website, survival analysis was automatically conducted, and the formula of miRNA prognostic signature was also directly obtained by OncomiR.

### FunRich Analysis

FunRich (http://www.funrich.org/) is a tool mainly used for functional enrichment and interaction network analysis of genes and proteins (Pathan and Keerthikumar, 2017), which was introduced to perform functional annotation and pathway enrichment for the target genes in this study.

### String Analysis

The protein–protein interaction (PPI) network analysis for target genes was performed by the STRING database (https://string-db.org/cgi/input.pl). This PPI network could be directly downloaded from the STRING database. Among all the protein–protein interactions, only those with score more than 0.4 were included for hub gene screening.

### Target Gene Prediction

The target genes of five potential miRNAs were predicted by a total of seven target gene prediction programs, involving PITA, RNA22, miRmap, microT, miRanda, PicTar, and TargetScan. To obtain more accurate analytic results, only target genes appeared in more than five target gene prediction databases were selected as the candidate target genes of miRNAs.

### Statistics Analysis

The statistics analyses in this study were automatically calculated by the online databases or tools as mentioned earlier. Continuous variables in normal distribution should be described as mean ± standard deviation (SD). Variance homogeneous and normal distributed continuous variables were compared by student *t*-test; otherwise, the Mann–Whitney U-test or Kruskal–Wallis H-test was used. *p*-value < 0.05 or log rank *p*-value < 0.05 was regarded as statistically significant.

## Data Availability

The original contributions presented in the study are included in the article/Supplementary Material; further inquiries can be directed to the corresponding authors.
