# Supplementary material for: A Comprehensive Analysis for Expression, Diagnosis, and Prognosis of m5C Regulator in Breast Cancer and Its ncRNA–mRNA Regulatory Mechanism
Source: Front Genet. 2022 Jun 22;13:822721. doi: 10.3389/fgene.2022.822721 (PMC9257136; doi:10.3389/fgene.2022.822721)
Supplement: Supplementary file 1 [file Table1.DOCX]

**Table S1**. The upstream miRNAs of DNMT3B or ALYREF predicted by starBase.

| miRNA | target gene |
| --- | --- |
| miR-29c-3p | DNMT3B |
| miR-30a-5p | DNMT3B |
| let-7b-5p | DNMT3B |
| let-7a-5p | DNMT3B |
| miR-375 | DNMT3B |
| miR-1-3p | DNMT3B |
| miR-135a-5p | DNMT3B |
| miR-195-5p | DNMT3B |
| miR-29a-3p | DNMT3B |
| miR-29b-3p | DNMT3B |
| miR-199a-5p | DNMT3B |
| miR-125a-5p | DNMT3B |
| miR-26a-5p | DNMT3B |
| miR-199b-5p | DNMT3B |
| miR-653-5p | DNMT3B |
| miR-26b-5p | DNMT3B |
| miR-656-3p | DNMT3B |
| miR-1251-5p | DNMT3B |
| miR-625-5p | DNMT3B |
| miR-668-3p | DNMT3B |
| miR-152-3p | DNMT3B |
| miR-510-5p | DNMT3B |
| miR-30c-5p | DNMT3B |
| miR-491-5p | DNMT3B |
| miR-6512-3p | DNMT3B |
| miR-665 | DNMT3B |
| let-7c-5p | DNMT3B |
| miR-499a-5p | DNMT3B |
| miR-432-5p | DNMT3B |
| miR-770-5p | DNMT3B |
| let-7f-5p | DNMT3B |
| miR-145-5p | DNMT3B |
| miR-494-3p | DNMT3B |
| miR-4306 | DNMT3B |
| miR-379-5p | DNMT3B |
| miR-497-5p | DNMT3B |
| miR-496 | DNMT3B |
| miR-3126-5p | DNMT3B |
| miR-1179 | DNMT3B |
| miR-367-3p | DNMT3B |
| miR-613 | DNMT3B |
| miR-620 | DNMT3B |
| miR-300 | DNMT3B |
| miR-1297 | DNMT3B |
| miR-125b-5p | DNMT3B |
| miR-4644 | DNMT3B |
| miR-5195-3p | DNMT3B |
| miR-543 | DNMT3B |
| miR-654-3p | DNMT3B |
| miR-200b-3p | DNMT3B |
| miR-335-5p | DNMT3B |
| miR-670-5p | DNMT3B |
| miR-874-3p | DNMT3B |
| miR-329-3p | DNMT3B |
| miR-124-3p | DNMT3B |
| miR-217 | DNMT3B |
| miR-299-3p | DNMT3B |
| miR-490-3p | DNMT3B |
| miR-3194-3p | DNMT3B |
| miR-506-3p | DNMT3B |
| miR-624-3p | DNMT3B |
| let-7e-5p | DNMT3B |
| miR-498 | DNMT3B |
| miR-556-5p | DNMT3B |
| miR-92b-3p | DNMT3B |
| miR-524-5p | DNMT3B |
| miR-650 | DNMT3B |
| miR-520d-5p | DNMT3B |
| miR-370-3p | DNMT3B |
| miR-381-3p | DNMT3B |
| miR-641 | DNMT3B |
| miR-493-3p | DNMT3B |
| miR-628-5p | DNMT3B |
| miR-3121-3p | DNMT3B |
| miR-5047 | DNMT3B |
| miR-760 | DNMT3B |
| miR-361-3p | DNMT3B |
| miR-2278 | DNMT3B |
| miR-363-3p | DNMT3B |
| miR-137 | DNMT3B |
| miR-95-3p | DNMT3B |
| miR-199a-3p | DNMT3B |
| miR-199b-3p | DNMT3B |
| miR-1913 | DNMT3B |
| miR-876-5p | DNMT3B |
| miR-3173-5p | DNMT3B |
| miR-431-5p | DNMT3B |
| miR-1323 | DNMT3B |
| miR-1270 | DNMT3B |
| miR-873-5p | DNMT3B |
| miR-129-2-3p | DNMT3B |
| miR-30d-5p | DNMT3B |
| miR-206 | DNMT3B |
| miR-15a-5p | DNMT3B |
| miR-129-1-3p | DNMT3B |
| miR-31-5p | DNMT3B |
| miR-30b-5p | DNMT3B |
| miR-148a-3p | DNMT3B |
| miR-429 | DNMT3B |
| miR-21-5p | DNMT3B |
| miR-885-5p | DNMT3B |
| miR-148b-3p | DNMT3B |
| miR-345-3p | DNMT3B |
| miR-4766-5p | DNMT3B |
| miR-339-5p | DNMT3B |
| miR-519d-3p | DNMT3B |
| miR-296-3p | DNMT3B |
| miR-328-3p | DNMT3B |
| miR-140-5p | DNMT3B |
| miR-222-3p | DNMT3B |
| let-7g-5p | DNMT3B |
| miR-362-3p | DNMT3B |
| miR-20b-5p | DNMT3B |
| miR-548o-3p | DNMT3B |
| miR-1343-3p | DNMT3B |
| miR-200c-3p | DNMT3B |
| miR-629-5p | DNMT3B |
| miR-651-5p | DNMT3B |
| let-7i-5p | DNMT3B |
| miR-16-5p | DNMT3B |
| miR-331-3p | DNMT3B |
| miR-580-3p | DNMT3B |
| miR-425-5p | DNMT3B |
| miR-30e-5p | DNMT3B |
| miR-18b-5p | DNMT3B |
| miR-455-5p | DNMT3B |
| miR-32-5p | DNMT3B |
| miR-183-5p | DNMT3B |
| miR-503-5p | DNMT3B |
| miR-340-5p | DNMT3B |
| miR-424-5p | DNMT3B |
| miR-4766-3p | DNMT3B |
| miR-185-5p | DNMT3B |
| miR-135b-5p | DNMT3B |
| miR-188-5p | DNMT3B |
| let-7d-5p | DNMT3B |
| miR-15b-5p | DNMT3B |
| miR-324-5p | DNMT3B |
| miR-2355-5p | DNMT3B |
| miR-1306-5p | DNMT3B |
| miR-106a-5p | DNMT3B |
| miR-98-5p | DNMT3B |
| miR-301b-3p | DNMT3B |
| miR-452-5p | DNMT3B |
| miR-934 | DNMT3B |
| miR-345-5p | DNMT3B |
| miR-324-3p | DNMT3B |
| miR-128-3p | DNMT3B |
| miR-1301-3p | DNMT3B |
| miR-20a-5p | DNMT3B |
| miR-92a-3p | DNMT3B |
| miR-590-5p | DNMT3B |
| miR-25-3p | DNMT3B |
| miR-93-5p | DNMT3B |
| miR-505-3p | DNMT3B |
| miR-17-5p | DNMT3B |
| miR-106b-5p | DNMT3B |
| miR-18a-5p | DNMT3B |
| miR-337-3p | ALYREF |
| miR-335-5p | ALYREF |
| miR-329-3p | ALYREF |
| miR-494-3p | ALYREF |
| miR-770-5p | ALYREF |
| miR-495-3p | ALYREF |
| miR-1321 | ALYREF |
| miR-3186-3p | ALYREF |
| miR-524-5p | ALYREF |
| miR-515-5p | ALYREF |
| miR-520d-5p | ALYREF |
| miR-552-3p | ALYREF |
| miR-1323 | ALYREF |
| miR-548o-3p | ALYREF |
| miR-18b-5p | ALYREF |
| miR-361-3p | ALYREF |
| miR-362-3p | ALYREF |
| miR-186-5p | ALYREF |
| miR-942-5p | ALYREF |
| miR-18a-5p | ALYREF |
