# Supplementary material for: A Comprehensive Analysis for Expression, Diagnosis, and Prognosis of m5C Regulator in Breast Cancer and Its ncRNA–mRNA Regulatory Mechanism
Source: Front Genet. 2022 Jun 22;13:822721. doi: 10.3389/fgene.2022.822721 (PMC9257136; doi:10.3389/fgene.2022.822721)
Supplement: Supplementary file 2 [file Table2.DOCX]

**Table S2**. The expression correlation between miRNA and mRNA in breast cancer determined by starBase.

| miRNA | gene | R | P-value |
| --- | --- | --- | --- |
| miR-29c-3p | DNMT3B | -0.34 | 9.36E-31 |
| miR-30a-5p | DNMT3B | -0.328 | 1.33E-28 |
| let-7b-5p | DNMT3B | -0.314 | 2.73E-26 |
| let-7a-5p | DNMT3B | -0.258 | 5.71E-18 |
| miR-375 | DNMT3B | -0.184 | 1.04E-09 |
| miR-1-3p | DNMT3B | -0.138 | 5.40E-06 |
| miR-135a-5p | DNMT3B | -0.135 | 8.25E-06 |
| miR-195-5p | DNMT3B | -0.119 | 9.02E-05 |
| miR-29a-3p | DNMT3B | -0.112 | 2.26E-04 |
| miR-29b-3p | DNMT3B | -0.106 | 4.51E-04 |
| miR-199a-5p | DNMT3B | -0.105 | 5.54E-04 |
| miR-125a-5p | DNMT3B | -0.097 | 1.43E-03 |
| miR-26a-5p | DNMT3B | -0.093 | 2.16E-03 |
| miR-199b-5p | DNMT3B | -0.091 | 2.68E-03 |
| miR-653-5p | DNMT3B | -0.078 | 9.92E-03 |
| miR-26b-5p | DNMT3B | -0.062 | 4.15E-02 |
| miR-656-3p | DNMT3B | -0.059 | 5.35E-02 |
| miR-1251-5p | DNMT3B | -0.057 | 5.93E-02 |
| miR-625-5p | DNMT3B | -0.054 | 7.46E-02 |
| miR-668-3p | DNMT3B | -0.048 | 1.15E-01 |
| miR-152-3p | DNMT3B | -0.045 | 1.40E-01 |
| miR-510-5p | DNMT3B | -0.042 | 1.67E-01 |
| miR-30c-5p | DNMT3B | -0.041 | 1.68E-01 |
| miR-491-5p | DNMT3B | -0.039 | 1.95E-01 |
| miR-6512-3p | DNMT3B | -0.036 | 2.33E-01 |
| miR-665 | DNMT3B | -0.035 | 2.52E-01 |
| let-7c-5p | DNMT3B | -0.034 | 2.68E-01 |
| miR-499a-5p | DNMT3B | -0.033 | 2.78E-01 |
| miR-432-5p | DNMT3B | -0.029 | 3.39E-01 |
| miR-770-5p | DNMT3B | -0.027 | 3.79E-01 |
| let-7f-5p | DNMT3B | -0.024 | 4.25E-01 |
| miR-145-5p | DNMT3B | -0.023 | 4.50E-01 |
| miR-494-3p | DNMT3B | -0.022 | 4.60E-01 |
| miR-4306 | DNMT3B | -0.017 | 5.78E-01 |
| miR-379-5p | DNMT3B | -0.016 | 6.02E-01 |
| miR-497-5p | DNMT3B | -0.015 | 6.17E-01 |
| miR-496 | DNMT3B | -0.014 | 6.43E-01 |
| miR-3126-5p | DNMT3B | -0.005 | 8.73E-01 |
| miR-1179 | DNMT3B | -0.003 | 9.14E-01 |
| miR-367-3p | DNMT3B | 0 | 1.00E+00 |
| miR-613 | DNMT3B | 0 | 1.00E+00 |
| miR-620 | DNMT3B | 0 | 1.00E+00 |
| miR-300 | DNMT3B | 0 | 1.00E+00 |
| miR-1297 | DNMT3B | 0 | 1.00E+00 |
| miR-125b-5p | DNMT3B | 0.002 | 9.47E-01 |
| miR-4644 | DNMT3B | 0.009 | 7.56E-01 |
| miR-5195-3p | DNMT3B | 0.01 | 7.39E-01 |
| miR-543 | DNMT3B | 0.011 | 7.06E-01 |
| miR-654-3p | DNMT3B | 0.012 | 6.87E-01 |
| miR-200b-3p | DNMT3B | 0.013 | 6.64E-01 |
| miR-335-5p | DNMT3B | 0.015 | 6.14E-01 |
| miR-670-5p | DNMT3B | 0.02 | 5.18E-01 |
| miR-874-3p | DNMT3B | 0.022 | 4.74E-01 |
| miR-329-3p | DNMT3B | 0.023 | 4.48E-01 |
| miR-124-3p | DNMT3B | 0.024 | 4.33E-01 |
| miR-217 | DNMT3B | 0.025 | 4.14E-01 |
| miR-299-3p | DNMT3B | 0.026 | 3.85E-01 |
| miR-490-3p | DNMT3B | 0.028 | 3.55E-01 |
| miR-3194-3p | DNMT3B | 0.033 | 2.78E-01 |
| miR-506-3p | DNMT3B | 0.035 | 2.46E-01 |
| miR-624-3p | DNMT3B | 0.035 | 2.44E-01 |
| let-7e-5p | DNMT3B | 0.037 | 2.21E-01 |
| miR-498 | DNMT3B | 0.039 | 2.02E-01 |
| miR-556-5p | DNMT3B | 0.039 | 2.05E-01 |
| miR-92b-3p | DNMT3B | 0.04 | 1.93E-01 |
| miR-524-5p | DNMT3B | 0.041 | 1.81E-01 |
| miR-650 | DNMT3B | 0.041 | 1.76E-01 |
| miR-520d-5p | DNMT3B | 0.042 | 1.62E-01 |
| miR-370-3p | DNMT3B | 0.043 | 1.53E-01 |
| miR-381-3p | DNMT3B | 0.045 | 1.42E-01 |
| miR-641 | DNMT3B | 0.046 | 1.26E-01 |
| miR-493-3p | DNMT3B | 0.047 | 1.24E-01 |
| miR-628-5p | DNMT3B | 0.052 | 8.52E-02 |
| miR-3121-3p | DNMT3B | 0.052 | 8.51E-02 |
| miR-5047 | DNMT3B | 0.052 | 8.63E-02 |
| miR-760 | DNMT3B | 0.055 | 7.22E-02 |
| miR-361-3p | DNMT3B | 0.058 | 5.67E-02 |
| miR-2278 | DNMT3B | 0.062 | 4.18E-02 |
| miR-363-3p | DNMT3B | 0.067 | 2.73E-02 |
| miR-137 | DNMT3B | 0.068 | 2.56E-02 |
| miR-95-3p | DNMT3B | 0.07 | 2.04E-02 |
| miR-199a-3p | DNMT3B | 0.07 | 2.12E-02 |
| miR-199b-3p | DNMT3B | 0.071 | 2.02E-02 |
| miR-1913 | DNMT3B | 0.072 | 1.71E-02 |
| miR-876-5p | DNMT3B | 0.077 | 1.08E-02 |
| miR-3173-5p | DNMT3B | 0.083 | 6.21E-03 |
| miR-431-5p | DNMT3B | 0.086 | 4.55E-03 |
| miR-1323 | DNMT3B | 0.093 | 2.23E-03 |
| miR-1270 | DNMT3B | 0.093 | 2.19E-03 |
| miR-873-5p | DNMT3B | 0.099 | 1.08E-03 |
| miR-129-2-3p | DNMT3B | 0.101 | 8.95E-04 |
| miR-30d-5p | DNMT3B | 0.104 | 5.77E-04 |
| miR-206 | DNMT3B | 0.105 | 5.29E-04 |
| miR-15a-5p | DNMT3B | 0.106 | 4.77E-04 |
| miR-129-1-3p | DNMT3B | 0.106 | 4.86E-04 |
| miR-31-5p | DNMT3B | 0.112 | 2.32E-04 |
| miR-30b-5p | DNMT3B | 0.112 | 2.19E-04 |
| miR-148a-3p | DNMT3B | 0.115 | 1.41E-04 |
| miR-429 | DNMT3B | 0.116 | 1.33E-04 |
| miR-21-5p | DNMT3B | 0.117 | 1.10E-04 |
| miR-885-5p | DNMT3B | 0.118 | 9.63E-05 |
| miR-148b-3p | DNMT3B | 0.122 | 5.47E-05 |
| miR-345-3p | DNMT3B | 0.122 | 5.70E-05 |
| miR-4766-5p | DNMT3B | 0.125 | 3.72E-05 |
| miR-339-5p | DNMT3B | 0.127 | 2.78E-05 |
| miR-519d-3p | DNMT3B | 0.127 | 2.89E-05 |
| miR-296-3p | DNMT3B | 0.137 | 6.31E-06 |
| miR-328-3p | DNMT3B | 0.139 | 4.55E-06 |
| miR-140-5p | DNMT3B | 0.145 | 1.66E-06 |
| miR-222-3p | DNMT3B | 0.152 | 4.95E-07 |
| let-7g-5p | DNMT3B | 0.156 | 2.51E-07 |
| miR-362-3p | DNMT3B | 0.158 | 1.58E-07 |
| miR-20b-5p | DNMT3B | 0.159 | 1.51E-07 |
| miR-548o-3p | DNMT3B | 0.163 | 6.45E-08 |
| miR-1343-3p | DNMT3B | 0.164 | 5.09E-08 |
| miR-200c-3p | DNMT3B | 0.165 | 4.27E-08 |
| miR-629-5p | DNMT3B | 0.167 | 3.16E-08 |
| miR-651-5p | DNMT3B | 0.171 | 1.54E-08 |
| let-7i-5p | DNMT3B | 0.176 | 5.86E-09 |
| miR-16-5p | DNMT3B | 0.178 | 3.66E-09 |
| miR-331-3p | DNMT3B | 0.178 | 3.45E-09 |
| miR-580-3p | DNMT3B | 0.194 | 1.07E-10 |
| miR-425-5p | DNMT3B | 0.196 | 7.43E-11 |
| miR-30e-5p | DNMT3B | 0.199 | 3.75E-11 |
| miR-18b-5p | DNMT3B | 0.202 | 1.93E-11 |
| miR-455-5p | DNMT3B | 0.202 | 1.78E-11 |
| miR-32-5p | DNMT3B | 0.216 | 6.47E-13 |
| miR-183-5p | DNMT3B | 0.235 | 4.78E-15 |
| miR-503-5p | DNMT3B | 0.236 | 3.59E-15 |
| miR-340-5p | DNMT3B | 0.237 | 2.36E-15 |
| miR-424-5p | DNMT3B | 0.244 | 3.39E-16 |
| miR-4766-3p | DNMT3B | 0.265 | 6.14E-19 |
| miR-185-5p | DNMT3B | 0.267 | 3.21E-19 |
| miR-135b-5p | DNMT3B | 0.274 | 3.91E-20 |
| miR-188-5p | DNMT3B | 0.276 | 1.76E-20 |
| let-7d-5p | DNMT3B | 0.28 | 5.64E-21 |
| miR-15b-5p | DNMT3B | 0.281 | 3.29E-21 |
| miR-324-5p | DNMT3B | 0.285 | 1.04E-21 |
| miR-2355-5p | DNMT3B | 0.291 | 1.37E-22 |
| miR-1306-5p | DNMT3B | 0.3 | 5.45E-24 |
| miR-106a-5p | DNMT3B | 0.307 | 3.55E-25 |
| miR-98-5p | DNMT3B | 0.316 | 1.27E-26 |
| miR-301b-3p | DNMT3B | 0.318 | 7.37E-27 |
| miR-452-5p | DNMT3B | 0.322 | 1.41E-27 |
| miR-934 | DNMT3B | 0.323 | 8.79E-28 |
| miR-345-5p | DNMT3B | 0.326 | 2.49E-28 |
| miR-324-3p | DNMT3B | 0.328 | 1.51E-28 |
| miR-128-3p | DNMT3B | 0.33 | 6.61E-29 |
| miR-1301-3p | DNMT3B | 0.365 | 1.90E-35 |
| miR-20a-5p | DNMT3B | 0.375 | 1.66E-37 |
| miR-92a-3p | DNMT3B | 0.396 | 4.96E-42 |
| miR-590-5p | DNMT3B | 0.398 | 1.74E-42 |
| miR-25-3p | DNMT3B | 0.414 | 2.93E-46 |
| miR-93-5p | DNMT3B | 0.448 | 1.42E-54 |
| miR-505-3p | DNMT3B | 0.457 | 3.61E-57 |
| miR-17-5p | DNMT3B | 0.458 | 2.47E-57 |
| miR-106b-5p | DNMT3B | 0.464 | 4.15E-59 |
| miR-18a-5p | DNMT3B | 0.484 | 8.76E-65 |
| miR-337-3p | ALYREF | -0.27 | 1.49E-19 |
| miR-335-5p | ALYREF | -0.096 | 1.60E-03 |
| miR-329-3p | ALYREF | -0.077 | 1.15E-02 |
| miR-494-3p | ALYREF | -0.076 | 1.21E-02 |
| miR-770-5p | ALYREF | -0.063 | 3.87E-02 |
| miR-495-3p | ALYREF | -0.043 | 1.61E-01 |
| miR-1321 | ALYREF | 0 | 1.00E+00 |
| miR-3186-3p | ALYREF | 0.039 | 1.95E-01 |
| miR-524-5p | ALYREF | 0.045 | 1.36E-01 |
| miR-515-5p | ALYREF | 0.061 | 4.30E-02 |
| miR-520d-5p | ALYREF | 0.068 | 2.45E-02 |
| miR-552-3p | ALYREF | 0.081 | 7.47E-03 |
| miR-1323 | ALYREF | 0.082 | 6.59E-03 |
| miR-548o-3p | ALYREF | 0.124 | 4.12E-05 |
| miR-18b-5p | ALYREF | 0.18 | 2.18E-09 |
| miR-361-3p | ALYREF | 0.189 | 3.78E-10 |
| miR-362-3p | ALYREF | 0.19 | 2.56E-10 |
| miR-186-5p | ALYREF | 0.226 | 5.34E-14 |
| miR-942-5p | ALYREF | 0.296 | 2.14E-23 |
| miR-18a-5p | ALYREF | 0.461 | 2.96E-58 |
