# Supplementary material for: A Comprehensive Analysis for Expression, Diagnosis, and Prognosis of m5C Regulator in Breast Cancer and Its ncRNA–mRNA Regulatory Mechanism
Source: Front Genet. 2022 Jun 22;13:822721. doi: 10.3389/fgene.2022.822721 (PMC9257136; doi:10.3389/fgene.2022.822721)
Supplement: Supplementary file 3 [file Table3.DOCX]

**Table S3**. The upstream lncRNAs of five potential miRNAs predicted by starBase and miRNet databases.

| miRNA | lncRNA |
| --- | --- |
| let-7b-5p | ZNF436-AS1 |
| let-7b-5p | SNHG12 |
| let-7b-5p | MIR29B2CHG |
| let-7b-5p | LINC01806 |
| let-7b-5p | LMCD1-AS1 |
| let-7b-5p | MUC20-OT1 |
| let-7b-5p | LINC00885 |
| let-7b-5p | LINC02432 |
| let-7b-5p | SLC9A3-AS1 |
| let-7b-5p | LINC02242 |
| let-7b-5p | SNHG4 |
| let-7b-5p | CARMN |
| let-7b-5p | HEIH |
| let-7b-5p | HCG18 |
| let-7b-5p | IER3-AS1 |
| let-7b-5p | HOXA11-AS |
| let-7b-5p | TRG-AS1 |
| let-7b-5p | LINC00265 |
| let-7b-5p | STAG3L5P-PVRIG2P-PILRB |
| let-7b-5p | CDKN2B-AS1 |
| let-7b-5p | LINC00963 |
| let-7b-5p | NUTM2A-AS1 |
| let-7b-5p | OLMALINC |
| let-7b-5p | RPARP-AS1 |
| let-7b-5p | LINC01001 |
| let-7b-5p | KCNQ1OT1 |
| let-7b-5p | LINC00294 |
| let-7b-5p | NEAT1 |
| let-7b-5p | LINC02381 |
| let-7b-5p | TMPO-AS1 |
| let-7b-5p | HELLPAR |
| let-7b-5p | G2E3-AS1 |
| let-7b-5p | VASH1-AS1 |
| let-7b-5p | MEG8 |
| let-7b-5p | OIP5-AS1 |
| let-7b-5p | IQCH-AS1 |
| let-7b-5p | DRAIC |
| let-7b-5p | THSD4-AS1 |
| let-7b-5p | UBL7-AS1 |
| let-7b-5p | ARHGAP27P1-BPTFP1-KPNA2P3 |
| let-7b-5p | SNHG16 |
| let-7b-5p | LINC01978 |
| let-7b-5p | TMEM147-AS1 |
| let-7b-5p | LINC00665 |
| let-7b-5p | ZNF571-AS1 |
| let-7b-5p | ZNF337-AS1 |
| let-7b-5p | MIR99AHG |
| let-7b-5p | LINC01678 |
| let-7b-5p | TTC28-AS1 |
| let-7b-5p | MIRLET7BHG |
| let-7b-5p | XIST |
| let-7b-5p | LINC00894 |
| let-7b-5p | TTTY15 |
| miR-195-5p | LINC01128 |
| miR-195-5p | TNFRSF14-AS1 |
| miR-195-5p | SNHG12 |
| miR-195-5p | SLFNL1-AS1 |
| miR-195-5p | ERI3-IT1 |
| miR-195-5p | RASAL2-AS1 |
| miR-195-5p | MIR29B2CHG |
| miR-195-5p | LINC01703 |
| miR-195-5p | PCBP1-AS1 |
| miR-195-5p | PAX8-AS1 |
| miR-195-5p | TEX41 |
| miR-195-5p | LINC02478 |
| miR-195-5p | CERS6-AS1 |
| miR-195-5p | TTN-AS1 |
| miR-195-5p | THUMPD3-AS1 |
| miR-195-5p | LINC00852 |
| miR-195-5p | FGD5-AS1 |
| miR-195-5p | LINC02035 |
| miR-195-5p | MBNL1-AS1 |
| miR-195-5p | DLG1-AS1 |
| miR-195-5p | AFAP1-AS1 |
| miR-195-5p | UBA6-AS1 |
| miR-195-5p | LINC02434 |
| miR-195-5p | SLC9A3-AS1 |
| miR-195-5p | BASP1-AS1 |
| miR-195-5p | EPB41L4A-AS1 |
| miR-195-5p | LINC01184 |
| miR-195-5p | HCG18 |
| miR-195-5p | HCG17 |
| miR-195-5p | TRAF3IP2-AS1 |
| miR-195-5p | LINC00473 |
| miR-195-5p | TRG-AS1 |
| miR-195-5p | DLX6-AS1 |
| miR-195-5p | STAG3L5P-PVRIG2P-PILRB |
| miR-195-5p | EXTL3-AS1 |
| miR-195-5p | CASC9 |
| miR-195-5p | AZIN1-AS1 |
| miR-195-5p | RNF139-AS1 |
| miR-195-5p | PVT1 |
| miR-195-5p | CDKN2B-AS1 |
| miR-195-5p | STX17-AS1 |
| miR-195-5p | GSN-AS1 |
| miR-195-5p | MIR600HG |
| miR-195-5p | SLC25A25-AS1 |
| miR-195-5p | C9orf163 |
| miR-195-5p | LINC00707 |
| miR-195-5p | PROSER2-AS1 |
| miR-195-5p | LINC00843 |
| miR-195-5p | BMS1P4 |
| miR-195-5p | ZSWIM8-AS1 |
| miR-195-5p | ZMIZ1-AS1 |
| miR-195-5p | NUTM2B-AS1 |
| miR-195-5p | NUTM2A-AS1 |
| miR-195-5p | KCNQ1OT1 |
| miR-195-5p | SNHG1 |
| miR-195-5p | NEAT1 |
| miR-195-5p | C1RL-AS1 |
| miR-195-5p | HOXC-AS3 |
| miR-195-5p | AGAP2-AS1 |
| miR-195-5p | HELLPAR |
| miR-195-5p | LINC00943 |
| miR-195-5p | TMEM132D-AS1 |
| miR-195-5p | ATXN8OS |
| miR-195-5p | LINC00641 |
| miR-195-5p | G2E3-AS1 |
| miR-195-5p | SLC25A21-AS1 |
| miR-195-5p | LINC00639 |
| miR-195-5p | PSMA3-AS1 |
| miR-195-5p | ACTN1-AS1 |
| miR-195-5p | MEG3 |
| miR-195-5p | MEG8 |
| miR-195-5p | LINC00638 |
| miR-195-5p | FAM30A |
| miR-195-5p | ARHGAP11B |
| miR-195-5p | GABPB1-AS1 |
| miR-195-5p | IQCH-AS1 |
| miR-195-5p | LINC01578 |
| miR-195-5p | ERVK13-1 |
| miR-195-5p | ZNF213-AS1 |
| miR-195-5p | SLX1B-SULT1A4 |
| miR-195-5p | LINC02128 |
| miR-195-5p | LINC00922 |
| miR-195-5p | LINC01572 |
| miR-195-5p | FENDRR |
| miR-195-5p | MIR497HG |
| miR-195-5p | LINC00324 |
| miR-195-5p | TBX2-AS1 |
| miR-195-5p | SNHG25 |
| miR-195-5p | SOX9-AS1 |
| miR-195-5p | LINC00511 |
| miR-195-5p | SNHG16 |
| miR-195-5p | TNRC6C-AS1 |
| miR-195-5p | PARD6G-AS1 |
| miR-195-5p | LINC00662 |
| miR-195-5p | SCGB1B2P |
| miR-195-5p | TMEM147-AS1 |
| miR-195-5p | LINC00665 |
| miR-195-5p | LIPE-AS1 |
| miR-195-5p | MZF1-AS1 |
| miR-195-5p | SMIM25 |
| miR-195-5p | LINC00649 |
| miR-195-5p | TRPM2-AS |
| miR-195-5p | TSPEAR-AS2 |
| miR-195-5p | LINC01547 |
| miR-195-5p | MCM3AP-AS1 |
| miR-195-5p | TUG1 |
| miR-195-5p | FAM239A |
| miR-195-5p | FAM239B |
| miR-195-5p | XIST |
| miR-195-5p | ARMCX5-GPRASP2 |
| miR-195-5p | MIR503HG |
| miR-195-5p | LINC00894 |
| miR-26a-5p | GAS5 |
| miR-26a-5p | MIR181A1HG |
| miR-26a-5p | LINC01703 |
| miR-26a-5p | THUMPD3-AS1 |
| miR-26a-5p | SH3BP5-AS1 |
| miR-26a-5p | DENND6A-DT |
| miR-26a-5p | PSMD6-AS2 |
| miR-26a-5p | NNT-AS1 |
| miR-26a-5p | LINC00847 |
| miR-26a-5p | HCG11 |
| miR-26a-5p | LINC00240 |
| miR-26a-5p | SNHG5 |
| miR-26a-5p | LINC00997 |
| miR-26a-5p | TRG-AS1 |
| miR-26a-5p | LINC00174 |
| miR-26a-5p | DLX6-AS1 |
| miR-26a-5p | LINC-PINT |
| miR-26a-5p | SNHG6 |
| miR-26a-5p | MSC-AS1 |
| miR-26a-5p | LINC01111 |
| miR-26a-5p | EBLN3P |
| miR-26a-5p | ENTPD1-AS1 |
| miR-26a-5p | RPARP-AS1 |
| miR-26a-5p | KCNQ1OT1 |
| miR-26a-5p | NEAT1 |
| miR-26a-5p | MALAT1 |
| miR-26a-5p | LINC00937 |
| miR-26a-5p | SNHG14 |
| miR-26a-5p | OIP5-AS1 |
| miR-26a-5p | GABPB1-AS1 |
| miR-26a-5p | WASIR2 |
| miR-26a-5p | MMP25-AS1 |
| miR-26a-5p | RRN3P2 |
| miR-26a-5p | ARHGAP27P1-BPTFP1-KPNA2P3 |
| miR-26a-5p | DLGAP1-AS1 |
| miR-26a-5p | DLGAP1-AS5 |
| miR-26a-5p | ZNF561-AS1 |
| miR-26a-5p | LINC00665 |
| miR-26a-5p | NORAD |
| miR-26a-5p | LINC00205 |
| miR-26a-5p | DUXAP8 |
| miR-26a-5p | MIATNB |
| miR-26a-5p | TUG1 |
| miR-26b-5p | GAS5 |
| miR-26b-5p | MIR181A1HG |
| miR-26b-5p | LINC01703 |
| miR-26b-5p | THUMPD3-AS1 |
| miR-26b-5p | SH3BP5-AS1 |
| miR-26b-5p | DENND6A-DT |
| miR-26b-5p | PSMD6-AS2 |
| miR-26b-5p | NNT-AS1 |
| miR-26b-5p | LINC00847 |
| miR-26b-5p | HCG11 |
| miR-26b-5p | LINC00240 |
| miR-26b-5p | SNHG5 |
| miR-26b-5p | LINC00997 |
| miR-26b-5p | TRG-AS1 |
| miR-26b-5p | LINC00174 |
| miR-26b-5p | DLX6-AS1 |
| miR-26b-5p | LINC-PINT |
| miR-26b-5p | SNHG6 |
| miR-26b-5p | MSC-AS1 |
| miR-26b-5p | LINC01111 |
| miR-26b-5p | EBLN3P |
| miR-26b-5p | ENTPD1-AS1 |
| miR-26b-5p | RPARP-AS1 |
| miR-26b-5p | KCNQ1OT1 |
| miR-26b-5p | NEAT1 |
| miR-26b-5p | MALAT1 |
| miR-26b-5p | LINC00937 |
| miR-26b-5p | SNHG14 |
| miR-26b-5p | OIP5-AS1 |
| miR-26b-5p | GABPB1-AS1 |
| miR-26b-5p | WASIR2 |
| miR-26b-5p | MMP25-AS1 |
| miR-26b-5p | RRN3P2 |
| miR-26b-5p | ARHGAP27P1-BPTFP1-KPNA2P3 |
| miR-26b-5p | DLGAP1-AS1 |
| miR-26b-5p | DLGAP1-AS5 |
| miR-26b-5p | ZNF561-AS1 |
| miR-26b-5p | LINC00665 |
| miR-26b-5p | NORAD |
| miR-26b-5p | LINC00205 |
| miR-26b-5p | DUXAP8 |
| miR-26b-5p | MIATNB |
| miR-26b-5p | TUG1 |
| miR-29a-3p | GAS5 |
| miR-29a-3p | MIR29B2CHG |
| miR-29a-3p | DNAJC27-AS1 |
| miR-29a-3p | PCBP1-AS1 |
| miR-29a-3p | LINC01907 |
| miR-29a-3p | THUMPD3-AS1 |
| miR-29a-3p | LINC00852 |
| miR-29a-3p | LINC00879 |
| miR-29a-3p | NOP14-AS1 |
| miR-29a-3p | MIR4458HG |
| miR-29a-3p | LIFR-AS1 |
| miR-29a-3p | HCG18 |
| miR-29a-3p | HCP5 |
| miR-29a-3p | AFDN-DT |
| miR-29a-3p | HOXA-AS3 |
| miR-29a-3p | HOXA10-AS |
| miR-29a-3p | SNHG15 |
| miR-29a-3p | STAG3L5P-PVRIG2P-PILRB |
| miR-29a-3p | LINC00689 |
| miR-29a-3p | PVT1 |
| miR-29a-3p | EBLN3P |
| miR-29a-3p | ARRDC1-AS1 |
| miR-29a-3p | H19 |
| miR-29a-3p | KCNQ1OT1 |
| miR-29a-3p | NEAT1 |
| miR-29a-3p | MIR4697HG |
| miR-29a-3p | LINC00943 |
| miR-29a-3p | VASH1-AS1 |
| miR-29a-3p | LINC00638 |
| miR-29a-3p | FAM30A |
| miR-29a-3p | RAD51-AS1 |
| miR-29a-3p | OIP5-AS1 |
| miR-29a-3p | DNAAF4-CCPG1 |
| miR-29a-3p | NPTN-IT1 |
| miR-29a-3p | LINC01578 |
| miR-29a-3p | MIR193BHG |
| miR-29a-3p | MIR762HG |
| miR-29a-3p | CRNDE |
| miR-29a-3p | MIR497HG |
| miR-29a-3p | CCDC144NL-AS1 |
| miR-29a-3p | LINC00511 |
| miR-29a-3p | SNHG20 |
| miR-29a-3p | LINC01224 |
| miR-29a-3p | SNHG17 |
| miR-29a-3p | LINC01270 |
| miR-29a-3p | MIR646HG |
| miR-29a-3p | DUXAP8 |
| miR-29a-3p | MIAT |
| miR-29a-3p | TUG1 |
| miR-29a-3p | LINC01521 |
| miR-29a-3p | MIRLET7BHG |
| miR-29a-3p | XIST |
