# Supplementary material for: A Comprehensive Analysis for Expression, Diagnosis, and Prognosis of m5C Regulator in Breast Cancer and Its ncRNA–mRNA Regulatory Mechanism
Source: Front Genet. 2022 Jun 22;13:822721. doi: 10.3389/fgene.2022.822721 (PMC9257136; doi:10.3389/fgene.2022.822721)
Supplement: Supplementary file 4 [file Table4.DOCX]

**Table S4**. The predicted target genes of 5 potential miRNAs.

| miRNA | Target |
| --- | --- |
| let-7b-5p | PTAFR |
| let-7b-5p | MED8 |
| let-7b-5p | USP24 |
| let-7b-5p | GNG5 |
| let-7b-5p | ZNF644 |
| let-7b-5p | NRAS |
| let-7b-5p | RGS16 |
| let-7b-5p | EDEM3 |
| let-7b-5p | PPP1R15B |
| let-7b-5p | YOD1 |
| let-7b-5p | AHCTF1 |
| let-7b-5p | PRDM2 |
| let-7b-5p | HOOK1 |
| let-7b-5p | PKN2 |
| let-7b-5p | PRPF38B |
| let-7b-5p | LRIG2 |
| let-7b-5p | SWT1 |
| let-7b-5p | SLC16A9 |
| let-7b-5p | CPEB3 |
| let-7b-5p | CCNJ |
| let-7b-5p | SLF2 |
| let-7b-5p | EIF4G2 |
| let-7b-5p | LGR4 |
| let-7b-5p | KCTD21 |
| let-7b-5p | RDX |
| let-7b-5p | ADAMTS8 |
| let-7b-5p | CLP1 |
| let-7b-5p | STX3 |
| let-7b-5p | NKAPD1 |
| let-7b-5p | LRIG3 |
| let-7b-5p | NAP1L1 |
| let-7b-5p | ATP2B1 |
| let-7b-5p | EEA1 |
| let-7b-5p | FGD6 |
| let-7b-5p | PLEKHG6 |
| let-7b-5p | HMGA2 |
| let-7b-5p | DYRK2 |
| let-7b-5p | ZNF10 |
| let-7b-5p | STARD13 |
| let-7b-5p | UFM1 |
| let-7b-5p | NHLRC3 |
| let-7b-5p | FNDC3A |
| let-7b-5p | RAB15 |
| let-7b-5p | ZFYVE26 |
| let-7b-5p | BEGAIN |
| let-7b-5p | NYNRIN |
| let-7b-5p | C14orf28 |
| let-7b-5p | SOCS4 |
| let-7b-5p | NAA30 |
| let-7b-5p | NIPA1 |
| let-7b-5p | KATNBL1 |
| let-7b-5p | RASGRP1 |
| let-7b-5p | GATM |
| let-7b-5p | ARPP19 |
| let-7b-5p | IGDCC3 |
| let-7b-5p | CPEB1 |
| let-7b-5p | C15orf41 |
| let-7b-5p | THBS1 |
| let-7b-5p | SNAP23 |
| let-7b-5p | MAPK6 |
| let-7b-5p | ARID3B |
| let-7b-5p | DNAJA2 |
| let-7b-5p | NME4 |
| let-7b-5p | EEF2K |
| let-7b-5p | GAN |
| let-7b-5p | CCR7 |
| let-7b-5p | COIL |
| let-7b-5p | INTS2 |
| let-7b-5p | LIMD2 |
| let-7b-5p | FAM104A |
| let-7b-5p | ARHGEF15 |
| let-7b-5p | NLK |
| let-7b-5p | CPD |
| let-7b-5p | ITGB3 |
| let-7b-5p | IGF2BP1 |
| let-7b-5p | ARHGAP28 |
| let-7b-5p | MIB1 |
| let-7b-5p | RIOK3 |
| let-7b-5p | GALNT1 |
| let-7b-5p | MYO1F |
| let-7b-5p | ZNF583 |
| let-7b-5p | E2F6 |
| let-7b-5p | SLC5A6 |
| let-7b-5p | MAP4K3 |
| let-7b-5p | MGAT4A |
| let-7b-5p | ACVR1C |
| let-7b-5p | FIGN |
| let-7b-5p | MYCN |
| let-7b-5p | RANBP2 |
| let-7b-5p | SLC20A1 |
| let-7b-5p | HOXD1 |
| let-7b-5p | COL3A1 |
| let-7b-5p | BZW1 |
| let-7b-5p | TTLL4 |
| let-7b-5p | GPCPD1 |
| let-7b-5p | ZNF512B |
| let-7b-5p | PARD6B |
| let-7b-5p | LTN1 |
| let-7b-5p | BACH1 |
| let-7b-5p | HIC2 |
| let-7b-5p | ANKRD28 |
| let-7b-5p | CDC25A |
| let-7b-5p | NME6 |
| let-7b-5p | FRMD4B |
| let-7b-5p | CD200R1 |
| let-7b-5p | IGF2BP2 |
| let-7b-5p | MASP1 |
| let-7b-5p | TRIM71 |
| let-7b-5p | PDE12 |
| let-7b-5p | CDV3 |
| let-7b-5p | FNDC3B |
| let-7b-5p | CASP3 |
| let-7b-5p | CPEB2 |
| let-7b-5p | SLC4A4 |
| let-7b-5p | FRAS1 |
| let-7b-5p | SMARCAD1 |
| let-7b-5p | USP38 |
| let-7b-5p | RICTOR |
| let-7b-5p | SLC38A9 |
| let-7b-5p | GCNT4 |
| let-7b-5p | FNIP1 |
| let-7b-5p | HAND1 |
| let-7b-5p | OSMR |
| let-7b-5p | C5orf51 |
| let-7b-5p | MAP3K1 |
| let-7b-5p | ADRB2 |
| let-7b-5p | PPARGC1B |
| let-7b-5p | SMIM3 |
| let-7b-5p | AC010441.1 |
| let-7b-5p | CPEB4 |
| let-7b-5p | TRIM41 |
| let-7b-5p | EDN1 |
| let-7b-5p | HMGA1 |
| let-7b-5p | SLC25A27 |
| let-7b-5p | LIN28B |
| let-7b-5p | RFX6 |
| let-7b-5p | UTRN |
| let-7b-5p | OSBPL3 |
| let-7b-5p | HOXA1 |
| let-7b-5p | CLDN12 |
| let-7b-5p | COL1A2 |
| let-7b-5p | AP1S1 |
| let-7b-5p | CPA4 |
| let-7b-5p | DLC1 |
| let-7b-5p | SNX16 |
| let-7b-5p | GDF6 |
| let-7b-5p | E2F5 |
| let-7b-5p | PTPRD |
| let-7b-5p | ZBTB5 |
| let-7b-5p | SLC35D2 |
| let-7b-5p | AL160269.1 |
| let-7b-5p | STRBP |
| let-7b-5p | PLPP6 |
| let-7b-5p | UHRF2 |
| let-7b-5p | ACER2 |
| let-7b-5p | HABP4 |
| let-7b-5p | TGFBR1 |
| let-7b-5p | PBX3 |
| let-7b-5p | DMD |
| let-7b-5p | PCDH19 |
| let-7b-5p | XK |
| let-7b-5p | PGRMC1 |
| let-7b-5p | INTS6L |
| let-7b-5p | ZNF275 |
| miR-195-5p | CC2D1B |
| miR-195-5p | SERBP1 |
| miR-195-5p | SYDE2 |
| miR-195-5p | CSDE1 |
| miR-195-5p | UBE2Q1 |
| miR-195-5p | ASH1L |
| miR-195-5p | HDGF |
| miR-195-5p | SIPA1L2 |
| miR-195-5p | AKT3 |
| miR-195-5p | UBE4B |
| miR-195-5p | KIF1B |
| miR-195-5p | SPEN |
| miR-195-5p | PDIK1L |
| miR-195-5p | ZNF362 |
| miR-195-5p | AGO1 |
| miR-195-5p | AK4 |
| miR-195-5p | SRSF11 |
| miR-195-5p | ADGRL2 |
| miR-195-5p | STXBP3 |
| miR-195-5p | GNAI3 |
| miR-195-5p | KCNC4 |
| miR-195-5p | LRIG2 |
| miR-195-5p | SUCO |
| miR-195-5p | C1orf21 |
| miR-195-5p | NAV1 |
| miR-195-5p | BTG2 |
| miR-195-5p | RASSF5 |
| miR-195-5p | WNT3A |
| miR-195-5p | CUL2 |
| miR-195-5p | CCDC6 |
| miR-195-5p | WAPL |
| miR-195-5p | CPEB3 |
| miR-195-5p | RAB11FIP2 |
| miR-195-5p | EIF3A |
| miR-195-5p | RET |
| miR-195-5p | PCGF5 |
| miR-195-5p | SLC35G1 |
| miR-195-5p | BTRC |
| miR-195-5p | SHOC2 |
| miR-195-5p | PLEKHA1 |
| miR-195-5p | STK33 |
| miR-195-5p | ARFGAP2 |
| miR-195-5p | SPTBN2 |
| miR-195-5p | CHORDC1 |
| miR-195-5p | BACE1 |
| miR-195-5p | SRPRA |
| miR-195-5p | WEE1 |
| miR-195-5p | ANO3 |
| miR-195-5p | ESRRA |
| miR-195-5p | ARL2 |
| miR-195-5p | CCND1 |
| miR-195-5p | SIDT2 |
| miR-195-5p | CHEK1 |
| miR-195-5p | VAMP1 |
| miR-195-5p | LRP6 |
| miR-195-5p | WBP11 |
| miR-195-5p | BHLHE41 |
| miR-195-5p | SINHCAF |
| miR-195-5p | TUBA1A |
| miR-195-5p | CSRNP2 |
| miR-195-5p | SPRYD3 |
| miR-195-5p | ZBTB39 |
| miR-195-5p | E2F7 |
| miR-195-5p | CDK17 |
| miR-195-5p | PRDM4 |
| miR-195-5p | ATXN2 |
| miR-195-5p | NAA25 |
| miR-195-5p | FBXO21 |
| miR-195-5p | CCND2 |
| miR-195-5p | PEX5 |
| miR-195-5p | EIF4B |
| miR-195-5p | HOXC11 |
| miR-195-5p | KIF5A |
| miR-195-5p | USP15 |
| miR-195-5p | LATS2 |
| miR-195-5p | DCLK1 |
| miR-195-5p | RUBCNL |
| miR-195-5p | PCDH9 |
| miR-195-5p | DACH1 |
| miR-195-5p | ZMYM2 |
| miR-195-5p | PCDH17 |
| miR-195-5p | FBXO33 |
| miR-195-5p | SOS2 |
| miR-195-5p | FERMT2 |
| miR-195-5p | ATG14 |
| miR-195-5p | ANGEL1 |
| miR-195-5p | BAG5 |
| miR-195-5p | CDCA4 |
| miR-195-5p | BCL2L2 |
| miR-195-5p | PPP2R5C |
| miR-195-5p | COPS2 |
| miR-195-5p | SPRED1 |
| miR-195-5p | CHAC1 |
| miR-195-5p | SEMA6D |
| miR-195-5p | FGF7 |
| miR-195-5p | RNF111 |
| miR-195-5p | FAM81A |
| miR-195-5p | MAP2K1 |
| miR-195-5p | LRRK1 |
| miR-195-5p | LITAF |
| miR-195-5p | SIAH1 |
| miR-195-5p | N4BP1 |
| miR-195-5p | SALL1 |
| miR-195-5p | DYNC1LI2 |
| miR-195-5p | BFAR |
| miR-195-5p | UBFD1 |
| miR-195-5p | RBBP6 |
| miR-195-5p | NFATC3 |
| miR-195-5p | PLA2G15 |
| miR-195-5p | BORCS6 |
| miR-195-5p | SLC25A35 |
| miR-195-5p | SYNRG |
| miR-195-5p | ATXN7L3 |
| miR-195-5p | RNF43 |
| miR-195-5p | AXIN2 |
| miR-195-5p | HELZ |
| miR-195-5p | ARHGDIA |
| miR-195-5p | PAFAH1B1 |
| miR-195-5p | TXNDC17 |
| miR-195-5p | CPD |
| miR-195-5p | CDK5R1 |
| miR-195-5p | PPM1D |
| miR-195-5p | KCNJ2 |
| miR-195-5p | GAREM1 |
| miR-195-5p | SMAD7 |
| miR-195-5p | BCL2 |
| miR-195-5p | KDSR |
| miR-195-5p | DSEL |
| miR-195-5p | USP14 |
| miR-195-5p | SEH1L |
| miR-195-5p | RNF125 |
| miR-195-5p | RNF138 |
| miR-195-5p | SOCS6 |
| miR-195-5p | INSR |
| miR-195-5p | MED26 |
| miR-195-5p | DYRK1B |
| miR-195-5p | KCNN4 |
| miR-195-5p | MAMSTR |
| miR-195-5p | FSD1 |
| miR-195-5p | CCNE1 |
| miR-195-5p | RAB4B |
| miR-195-5p | MIA-RAB4B |
| miR-195-5p | AP2A1 |
| miR-195-5p | PPP2R1A |
| miR-195-5p | MYT1L |
| miR-195-5p | LMAN2L |
| miR-195-5p | MGAT4A |
| miR-195-5p | LRP1B |
| miR-195-5p | COBLL1 |
| miR-195-5p | LRP2 |
| miR-195-5p | TLK1 |
| miR-195-5p | IHH |
| miR-195-5p | PID1 |
| miR-195-5p | RAB10 |
| miR-195-5p | NRBP1 |
| miR-195-5p | CRIM1 |
| miR-195-5p | ACTR2 |
| miR-195-5p | CCNT2 |
| miR-195-5p | ACVR2A |
| miR-195-5p | KIF5C |
| miR-195-5p | UBR3 |
| miR-195-5p | MOB4 |
| miR-195-5p | STRADB |
| miR-195-5p | 2-Sep |
| miR-195-5p | TMEM189-UBE2V1 |
| miR-195-5p | UBE2V1 |
| miR-195-5p | SALL4 |
| miR-195-5p | ZBTB46 |
| miR-195-5p | ZCCHC3 |
| miR-195-5p | POLR3F |
| miR-195-5p | SYNDIG1 |
| miR-195-5p | TGIF2 |
| miR-195-5p | ZSWIM3 |
| miR-195-5p | ADAMTS5 |
| miR-195-5p | SYNJ1 |
| miR-195-5p | USP25 |
| miR-195-5p | PISD |
| miR-195-5p | YWHAH |
| miR-195-5p | NUP50 |
| miR-195-5p | RAF1 |
| miR-195-5p | SLC4A7 |
| miR-195-5p | TRANK1 |
| miR-195-5p | LRRFIP2 |
| miR-195-5p | HIGD1A |
| miR-195-5p | CDC25A |
| miR-195-5p | DENND6A |
| miR-195-5p | RYBP |
| miR-195-5p | BTLA |
| miR-195-5p | ATP13A3 |
| miR-195-5p | PDCD6IP |
| miR-195-5p | SNRK |
| miR-195-5p | RBM6 |
| miR-195-5p | FRYL |
| miR-195-5p | KDR |
| miR-195-5p | CNOT6L |
| miR-195-5p | TSPAN5 |
| miR-195-5p | FBXW7 |
| miR-195-5p | CPEB2 |
| miR-195-5p | DCUN1D4 |
| miR-195-5p | FGF2 |
| miR-195-5p | HSPA4L |
| miR-195-5p | NAA15 |
| miR-195-5p | TLL1 |
| miR-195-5p | STOX2 |
| miR-195-5p | AFF4 |
| miR-195-5p | CCNJL |
| miR-195-5p | ITGA2 |
| miR-195-5p | SLC12A2 |
| miR-195-5p | ISOC1 |
| miR-195-5p | SMAD5 |
| miR-195-5p | PURA |
| miR-195-5p | ADRB2 |
| miR-195-5p | TENM2 |
| miR-195-5p | WWC1 |
| miR-195-5p | CREBRF |
| miR-195-5p | SRPK1 |
| miR-195-5p | UBE2J1 |
| miR-195-5p | EPHA7 |
| miR-195-5p | GPR63 |
| miR-195-5p | PNISR |
| miR-195-5p | CD164 |
| miR-195-5p | MAP7 |
| miR-195-5p | PLAGL1 |
| miR-195-5p | DLL1 |
| miR-195-5p | JARID2 |
| miR-195-5p | RNF144B |
| miR-195-5p | E2F3 |
| miR-195-5p | HMGA1 |
| miR-195-5p | PIM1 |
| miR-195-5p | VEGFA |
| miR-195-5p | CD2AP |
| miR-195-5p | SH3BGRL2 |
| miR-195-5p | RNF217 |
| miR-195-5p | RSPO3 |
| miR-195-5p | TBPL1 |
| miR-195-5p | MYB |
| miR-195-5p | PHACTR2 |
| miR-195-5p | KBTBD2 |
| miR-195-5p | SEMA3A |
| miR-195-5p | SEMA3D |
| miR-195-5p | PDK4 |
| miR-195-5p | SMURF1 |
| miR-195-5p | RELN |
| miR-195-5p | SYPL1 |
| miR-195-5p | KMT2C |
| miR-195-5p | WIPI2 |
| miR-195-5p | AVL9 |
| miR-195-5p | DMTF1 |
| miR-195-5p | CLDN12 |
| miR-195-5p | ANKIB1 |
| miR-195-5p | DYNC1I1 |
| miR-195-5p | CAPZA2 |
| miR-195-5p | AHCYL2 |
| miR-195-5p | ZYX |
| miR-195-5p | FGFR1 |
| miR-195-5p | SLC20A2 |
| miR-195-5p | PLAG1 |
| miR-195-5p | MYBL1 |
| miR-195-5p | EYA1 |
| miR-195-5p | SNX16 |
| miR-195-5p | RUNX1T1 |
| miR-195-5p | HAS2 |
| miR-195-5p | ZHX1 |
| miR-195-5p | SLC7A2 |
| miR-195-5p | XPO7 |
| miR-195-5p | WWP1 |
| miR-195-5p | FAM91A1 |
| miR-195-5p | PTPRD |
| miR-195-5p | PTCH1 |
| miR-195-5p | PHF19 |
| miR-195-5p | GOLGA1 |
| miR-195-5p | PPP6C |
| miR-195-5p | CDC37L1 |
| miR-195-5p | RAD23B |
| miR-195-5p | PAPPA |
| miR-195-5p | ZBTB34 |
| miR-195-5p | DOLPP1 |
| miR-195-5p | RPS6KA3 |
| miR-195-5p | ARMCX2 |
| miR-195-5p | ACSL4 |
| miR-195-5p | TMEM255A |
| miR-195-5p | APLN |
| miR-195-5p | RAP2C |
| miR-195-5p | DDX3X |
| miR-195-5p | CLCN5 |
| miR-195-5p | TSPYL2 |
| miR-195-5p | ZNF449 |
| miR-195-5p | SLC9A6 |
| miR-29a-3p | MEGF6 |
| miR-29a-3p | CTNNBIP1 |
| miR-29a-3p | EPS15 |
| miR-29a-3p | ANKRD13C |
| miR-29a-3p | COL11A1 |
| miR-29a-3p | SLC16A1 |
| miR-29a-3p | SIKE1 |
| miR-29a-3p | PRKAB2 |
| miR-29a-3p | MCL1 |
| miR-29a-3p | LYSMD1 |
| miR-29a-3p | CLK2 |
| miR-29a-3p | KDM5B |
| miR-29a-3p | PPP1R15B |
| miR-29a-3p | CDC42BPA |
| miR-29a-3p | AKT3 |
| miR-29a-3p | SH3BP5L |
| miR-29a-3p | CDC42 |
| miR-29a-3p | PDIK1L |
| miR-29a-3p | ZNF362 |
| miR-29a-3p | RLF |
| miR-29a-3p | NASP |
| miR-29a-3p | CMPK1 |
| miR-29a-3p | GPX7 |
| miR-29a-3p | NFIA |
| miR-29a-3p | SYPL2 |
| miR-29a-3p | SETDB1 |
| miR-29a-3p | KIRREL1 |
| miR-29a-3p | TMEM183A |
| miR-29a-3p | DIP2C |
| miR-29a-3p | LARP4B |
| miR-29a-3p | ITGB1 |
| miR-29a-3p | RHOBTB1 |
| miR-29a-3p | FRAT2 |
| miR-29a-3p | ADAM12 |
| miR-29a-3p | TET1 |
| miR-29a-3p | CCSER2 |
| miR-29a-3p | PTEN |
| miR-29a-3p | ENTPD7 |
| miR-29a-3p | TAF5 |
| miR-29a-3p | SPTY2D1 |
| miR-29a-3p | NUP160 |
| miR-29a-3p | OSBP |
| miR-29a-3p | KMT5B |
| miR-29a-3p | RAB30 |
| miR-29a-3p | PGAP2 |
| miR-29a-3p | ZDHHC5 |
| miR-29a-3p | NKAPD1 |
| miR-29a-3p | TNFRSF1A |
| miR-29a-3p | YBX3 |
| miR-29a-3p | LRP6 |
| miR-29a-3p | COL2A1 |
| miR-29a-3p | NCKAP5L |
| miR-29a-3p | PAN2 |
| miR-29a-3p | IGF1 |
| miR-29a-3p | FOXJ2 |
| miR-29a-3p | NAV3 |
| miR-29a-3p | TMTC3 |
| miR-29a-3p | TDG |
| miR-29a-3p | N4BP2L1 |
| miR-29a-3p | PROSER1 |
| miR-29a-3p | KDELC1 |
| miR-29a-3p | COL4A1 |
| miR-29a-3p | DGKH |
| miR-29a-3p | COL4A2 |
| miR-29a-3p | VCPKMT |
| miR-29a-3p | FERMT2 |
| miR-29a-3p | GMFB |
| miR-29a-3p | ZFP36L1 |
| miR-29a-3p | DIO2 |
| miR-29a-3p | EML5 |
| miR-29a-3p | PPP1R13B |
| miR-29a-3p | ABHD4 |
| miR-29a-3p | TTC9 |
| miR-29a-3p | KIF26A |
| miR-29a-3p | BMF |
| miR-29a-3p | RFX7 |
| miR-29a-3p | MEX3B |
| miR-29a-3p | CTDSPL2 |
| miR-29a-3p | AP4E1 |
| miR-29a-3p | TPM1 |
| miR-29a-3p | FEM1B |
| miR-29a-3p | IREB2 |
| miR-29a-3p | MTSS1L |
| miR-29a-3p | AP1G1 |
| miR-29a-3p | KCTD5 |
| miR-29a-3p | GLIS2 |
| miR-29a-3p | C16orf72 |
| miR-29a-3p | MAZ |
| miR-29a-3p | HAS3 |
| miR-29a-3p | PMP22 |
| miR-29a-3p | UBTF |
| miR-29a-3p | COL1A1 |
| miR-29a-3p | MBTD1 |
| miR-29a-3p | SMTNL2 |
| miR-29a-3p | KDM6B |
| miR-29a-3p | HS3ST3B1 |
| miR-29a-3p | ANKRD13B |
| miR-29a-3p | YPEL2 |
| miR-29a-3p | PPM1D |
| miR-29a-3p | RNF138 |
| miR-29a-3p | COL5A3 |
| miR-29a-3p | IFI30 |
| miR-29a-3p | AC007192.1 |
| miR-29a-3p | KIAA0355 |
| miR-29a-3p | HIF3A |
| miR-29a-3p | KMT5C |
| miR-29a-3p | PXDN |
| miR-29a-3p | ATAD2B |
| miR-29a-3p | DNMT3A |
| miR-29a-3p | SLC30A3 |
| miR-29a-3p | PPP4R3B |
| miR-29a-3p | BCL11A |
| miR-29a-3p | FAM136A |
| miR-29a-3p | SESTD1 |
| miR-29a-3p | COL5A2 |
| miR-29a-3p | USP37 |
| miR-29a-3p | ABCB6 |
| miR-29a-3p | AC068946.2 |
| miR-29a-3p | ATG9A |
| miR-29a-3p | WDFY1 |
| miR-29a-3p | COL4A4 |
| miR-29a-3p | SLC16A14 |
| miR-29a-3p | COL6A3 |
| miR-29a-3p | HDAC4 |
| miR-29a-3p | TRIB2 |
| miR-29a-3p | MYCN |
| miR-29a-3p | RMND5A |
| miR-29a-3p | CCNT2 |
| miR-29a-3p | ACVR2A |
| miR-29a-3p | COL3A1 |
| miR-29a-3p | CCNYL1 |
| miR-29a-3p | TMEM169 |
| miR-29a-3p | COL4A3 |
| miR-29a-3p | DGKD |
| miR-29a-3p | MAFB |
| miR-29a-3p | ZBTB46 |
| miR-29a-3p | DNMT3B |
| miR-29a-3p | NCOA3 |
| miR-29a-3p | TFAP2C |
| miR-29a-3p | SS18L1 |
| miR-29a-3p | TIAM1 |
| miR-29a-3p | BRWD1 |
| miR-29a-3p | TMPRSS3 |
| miR-29a-3p | ARVCF |
| miR-29a-3p | C1QTNF6 |
| miR-29a-3p | CCDC117 |
| miR-29a-3p | SMARCC1 |
| miR-29a-3p | COL7A1 |
| miR-29a-3p | ADAMTS9 |
| miR-29a-3p | FSTL1 |
| miR-29a-3p | COMMD2 |
| miR-29a-3p | CLDN1 |
| miR-29a-3p | GXYLT2 |
| miR-29a-3p | SIDT1 |
| miR-29a-3p | PLXNA1 |
| miR-29a-3p | MLF1 |
| miR-29a-3p | IL1RAP |
| miR-29a-3p | ELF2 |
| miR-29a-3p | OTUD4 |
| miR-29a-3p | PDGFC |
| miR-29a-3p | FRAS1 |
| miR-29a-3p | FAM241A |
| miR-29a-3p | ABCE1 |
| miR-29a-3p | TLL1 |
| miR-29a-3p | HMGCS1 |
| miR-29a-3p | ARRDC3 |
| miR-29a-3p | PPIC |
| miR-29a-3p | FAM13B |
| miR-29a-3p | HBEGF |
| miR-29a-3p | SPARC |
| miR-29a-3p | UBTD2 |
| miR-29a-3p | ISL1 |
| miR-29a-3p | PIK3R1 |
| miR-29a-3p | SNX24 |
| miR-29a-3p | PURA |
| miR-29a-3p | PCDHA6 |
| miR-29a-3p | PCDHA9 |
| miR-29a-3p | PCDHA8 |
| miR-29a-3p | PCDHA7 |
| miR-29a-3p | PCDHA5 |
| miR-29a-3p | PCDHA4 |
| miR-29a-3p | PCDHA2 |
| miR-29a-3p | PCDHA1 |
| miR-29a-3p | PCDHA13 |
| miR-29a-3p | PCDHAC2 |
| miR-29a-3p | PCDHAC1 |
| miR-29a-3p | PCDHA11 |
| miR-29a-3p | PCDHA10 |
| miR-29a-3p | PCDHA12 |
| miR-29a-3p | PCDHA3 |
| miR-29a-3p | MFAP3 |
| miR-29a-3p | LSM11 |
| miR-29a-3p | TUBB2A |
| miR-29a-3p | BAK1 |
| miR-29a-3p | TFEB |
| miR-29a-3p | XPO5 |
| miR-29a-3p | GSTA4 |
| miR-29a-3p | HMGN3 |
| miR-29a-3p | ELOVL4 |
| miR-29a-3p | REV3L |
| miR-29a-3p | JARID2 |
| miR-29a-3p | KLHDC3 |
| miR-29a-3p | VEGFA |
| miR-29a-3p | PTP4A1 |
| miR-29a-3p | COL19A1 |
| miR-29a-3p | BMT2 |
| miR-29a-3p | TFEC |
| miR-29a-3p | GPR37 |
| miR-29a-3p | ELN |
| miR-29a-3p | COL1A2 |
| miR-29a-3p | HBP1 |
| miR-29a-3p | CAV2 |
| miR-29a-3p | ARF5 |
| miR-29a-3p | MEST |
| miR-29a-3p | ZNF282 |
| miR-29a-3p | INSIG1 |
| miR-29a-3p | RNF19A |
| miR-29a-3p | TMEM65 |
| miR-29a-3p | LPL |
| miR-29a-3p | PI15 |
| miR-29a-3p | CRISPLD1 |
| miR-29a-3p | ZBTB10 |
| miR-29a-3p | ZBTB5 |
| miR-29a-3p | KLF4 |
| miR-29a-3p | COL15A1 |
| miR-29a-3p | ZBTB34 |
| miR-29a-3p | SPTAN1 |
| miR-29a-3p | COL5A1 |
| miR-29a-3p | SCML2 |
| miR-29a-3p | AMER1 |
| miR-29a-3p | NEXMIF |
| miR-29a-3p | BRWD3 |
| miR-29a-3p | NKRF |
| miR-29a-3p | SHROOM2 |
| miR-29a-3p | DDX3X |
| miR-29a-3p | STARD8 |
| miR-29a-3p | COL4A5 |
| miR-26a-5p | CASZ1 |
| miR-26a-5p | ATPAF1 |
| miR-26a-5p | SLC19A2 |
| miR-26a-5p | TTC13 |
| miR-26a-5p | ERO1B |
| miR-26a-5p | ATP1A2 |
| miR-26a-5p | CEP350 |
| miR-26a-5p | LARP4B |
| miR-26a-5p | ACBD5 |
| miR-26a-5p | CCDC6 |
| miR-26a-5p | SLC25A16 |
| miR-26a-5p | DLG5 |
| miR-26a-5p | FRAT2 |
| miR-26a-5p | ZDHHC6 |
| miR-26a-5p | EIF3A |
| miR-26a-5p | REEP3 |
| miR-26a-5p | TET1 |
| miR-26a-5p | PTEN |
| miR-26a-5p | MXI1 |
| miR-26a-5p | EIF4G2 |
| miR-26a-5p | CREBZF |
| miR-26a-5p | CHORDC1 |
| miR-26a-5p | RCN1 |
| miR-26a-5p | PRR5L |
| miR-26a-5p | OAF |
| miR-26a-5p | SLC38A2 |
| miR-26a-5p | ZFC3H1 |
| miR-26a-5p | PAWR |
| miR-26a-5p | ANKS1B |
| miR-26a-5p | HECTD4 |
| miR-26a-5p | CDK2AP1 |
| miR-26a-5p | KIAA1551 |
| miR-26a-5p | LARP4 |
| miR-26a-5p | USP15 |
| miR-26a-5p | ULK1 |
| miR-26a-5p | LNX2 |
| miR-26a-5p | MAB21L1 |
| miR-26a-5p | GNPNAT1 |
| miR-26a-5p | STYX |
| miR-26a-5p | PELI2 |
| miR-26a-5p | PLEKHH1 |
| miR-26a-5p | CPSF2 |
| miR-26a-5p | EIF5 |
| miR-26a-5p | MEX3B |
| miR-26a-5p | FAM98B |
| miR-26a-5p | CHAC1 |
| miR-26a-5p | USP3 |
| miR-26a-5p | RCN2 |
| miR-26a-5p | UBE2G1 |
| miR-26a-5p | ULK2 |
| miR-26a-5p | LSM12 |
| miR-26a-5p | TOB1 |
| miR-26a-5p | SLC16A6 |
| miR-26a-5p | NLK |
| miR-26a-5p | DCAF7 |
| miR-26a-5p | PITPNC1 |
| miR-26a-5p | C18orf25 |
| miR-26a-5p | FAM98A |
| miR-26a-5p | FAM136A |
| miR-26a-5p | STK39 |
| miR-26a-5p | ATF2 |
| miR-26a-5p | LTBP1 |
| miR-26a-5p | INHBB |
| miR-26a-5p | MTX2 |
| miR-26a-5p | NAB1 |
| miR-26a-5p | STRADB |
| miR-26a-5p | C20orf24 |
| miR-26a-5p | DYRK1A |
| miR-26a-5p | TMEM184B |
| miR-26a-5p | CLASP2 |
| miR-26a-5p | FRMD4B |
| miR-26a-5p | OSBPL11 |
| miR-26a-5p | RYK |
| miR-26a-5p | PLOD2 |
| miR-26a-5p | SERP1 |
| miR-26a-5p | PDCD10 |
| miR-26a-5p | NCEH1 |
| miR-26a-5p | BHLHE40 |
| miR-26a-5p | PRKCD |
| miR-26a-5p | CD200 |
| miR-26a-5p | SENP5 |
| miR-26a-5p | NAP1L5 |
| miR-26a-5p | LEF1 |
| miR-26a-5p | PGRMC2 |
| miR-26a-5p | SLC7A11 |
| miR-26a-5p | INTU |
| miR-26a-5p | NAA15 |
| miR-26a-5p | SMAD1 |
| miR-26a-5p | FAM160A1 |
| miR-26a-5p | ETF1 |
| miR-26a-5p | ADAM19 |
| miR-26a-5p | CCNJL |
| miR-26a-5p | TNPO1 |
| miR-26a-5p | SRP19 |
| miR-26a-5p | LARP1 |
| miR-26a-5p | LSM11 |
| miR-26a-5p | CREBRF |
| miR-26a-5p | HMGA1 |
| miR-26a-5p | ANKS1A |
| miR-26a-5p | PHF3 |
| miR-26a-5p | PNRC1 |
| miR-26a-5p | NUS1 |
| miR-26a-5p | CCDC28A |
| miR-26a-5p | HOXA5 |
| miR-26a-5p | DOCK4 |
| miR-26a-5p | EZH2 |
| miR-26a-5p | KMT2C |
| miR-26a-5p | CDK13 |
| miR-26a-5p | MFHAS1 |
| miR-26a-5p | REEP4 |
| miR-26a-5p | TP53INP1 |
| miR-26a-5p | PHF20L1 |
| miR-26a-5p | B4GALT1 |
| miR-26a-5p | ASPN |
| miR-26a-5p | STRBP |
| miR-26a-5p | CAMSAP1 |
| miR-26a-5p | ZNF462 |
| miR-26a-5p | WNK3 |
| miR-26a-5p | ATP11C |
| miR-26a-5p | NHS |
| miR-26a-5p | DDX3X |
| miR-26b-5p | CASZ1 |
| miR-26b-5p | ATPAF1 |
| miR-26b-5p | TTC13 |
| miR-26b-5p | NID1 |
| miR-26b-5p | ERO1B |
| miR-26b-5p | ATP1A2 |
| miR-26b-5p | CEP350 |
| miR-26b-5p | RHOU |
| miR-26b-5p | LARP4B |
| miR-26b-5p | ACBD5 |
| miR-26b-5p | CCDC6 |
| miR-26b-5p | SLC25A16 |
| miR-26b-5p | DLG5 |
| miR-26b-5p | ZDHHC6 |
| miR-26b-5p | EIF3A |
| miR-26b-5p | REEP3 |
| miR-26b-5p | TET1 |
| miR-26b-5p | PTEN |
| miR-26b-5p | MXI1 |
| miR-26b-5p | EIF4G2 |
| miR-26b-5p | CREBZF |
| miR-26b-5p | CHORDC1 |
| miR-26b-5p | SLC38A2 |
| miR-26b-5p | ZFC3H1 |
| miR-26b-5p | PAWR |
| miR-26b-5p | ANKS1B |
| miR-26b-5p | HECTD4 |
| miR-26b-5p | CDK2AP1 |
| miR-26b-5p | KLHL42 |
| miR-26b-5p | KIAA1551 |
| miR-26b-5p | LARP4 |
| miR-26b-5p | USP15 |
| miR-26b-5p | ULK1 |
| miR-26b-5p | LNX2 |
| miR-26b-5p | MAB21L1 |
| miR-26b-5p | CDK8 |
| miR-26b-5p | GNPNAT1 |
| miR-26b-5p | STYX |
| miR-26b-5p | PELI2 |
| miR-26b-5p | PLEKHH1 |
| miR-26b-5p | CPSF2 |
| miR-26b-5p | EIF5 |
| miR-26b-5p | MEX3B |
| miR-26b-5p | FAM98B |
| miR-26b-5p | USP3 |
| miR-26b-5p | RCN2 |
| miR-26b-5p | UBE2G1 |
| miR-26b-5p | ULK2 |
| miR-26b-5p | LSM12 |
| miR-26b-5p | TOB1 |
| miR-26b-5p | NLK |
| miR-26b-5p | DCAF7 |
| miR-26b-5p | PITPNC1 |
| miR-26b-5p | C18orf25 |
| miR-26b-5p | FAM98A |
| miR-26b-5p | FAM136A |
| miR-26b-5p | STK39 |
| miR-26b-5p | ATF2 |
| miR-26b-5p | MTX2 |
| miR-26b-5p | NAB1 |
| miR-26b-5p | STRADB |
| miR-26b-5p | C20orf24 |
| miR-26b-5p | DYRK1A |
| miR-26b-5p | TMEM184B |
| miR-26b-5p | CLASP2 |
| miR-26b-5p | FRMD4B |
| miR-26b-5p | OSBPL11 |
| miR-26b-5p | PLOD2 |
| miR-26b-5p | SERP1 |
| miR-26b-5p | PDCD10 |
| miR-26b-5p | NCEH1 |
| miR-26b-5p | BHLHE40 |
| miR-26b-5p | PRKCD |
| miR-26b-5p | NAP1L5 |
| miR-26b-5p | LEF1 |
| miR-26b-5p | SLC7A11 |
| miR-26b-5p | INTU |
| miR-26b-5p | SMAD1 |
| miR-26b-5p | FAM160A1 |
| miR-26b-5p | ETF1 |
| miR-26b-5p | ADAM19 |
| miR-26b-5p | TNPO1 |
| miR-26b-5p | SRP19 |
| miR-26b-5p | LARP1 |
| miR-26b-5p | CREBRF |
| miR-26b-5p | TFAP2A |
| miR-26b-5p | HMGA1 |
| miR-26b-5p | PNRC1 |
| miR-26b-5p | NUS1 |
| miR-26b-5p | CCDC28A |
| miR-26b-5p | HOXA5 |
| miR-26b-5p | DOCK4 |
| miR-26b-5p | EZH2 |
| miR-26b-5p | KMT2C |
| miR-26b-5p | MFHAS1 |
| miR-26b-5p | B4GALT1 |
| miR-26b-5p | ASPN |
| miR-26b-5p | STRBP |
| miR-26b-5p | CAMSAP1 |
| miR-26b-5p | ZNF462 |
| miR-26b-5p | WNK3 |
| miR-26b-5p | ATP11C |
| miR-26b-5p | NHS |
| miR-26b-5p | DDX3X |
